# The methylation profile of *IL4*, *IL5*, *IL10*, *IFNG* and *FOXP3* associated with environmental exposures differed between Polish infants with the food allergy and/or atopic dermatitis and without the disease

**DOI:** 10.3389/fimmu.2023.1209190

**Published:** 2023-07-13

**Authors:** Marta Gorzkiewicz, Ewa Łoś-Rycharska, Julia Gawryjołek, Marcin Gołębiewski, Aneta Krogulska, Tomasz Grzybowski

**Affiliations:** ^1^ Department of Forensic Medicine, Ludwik Rydygier Collegium Medicum in Bydgoszcz, Nicolaus Copernicus University in Toruń, Toruń, Poland; ^2^ Department of Pediatrics, Allergology and Gastroenterology, Ludwik Rydygier Collegium Medicum in Bydgoszcz, Nicolaus Copernicus University in Toruń, Toruń, Poland; ^3^ Department of Plant Physiology and Biotechnology, Nicolaus Copernicus University in Toruń, Toruń, Poland; ^4^ Interdisciplinary Centre of Modern Technologies, Nicolaus Copernicus University in Toruń, Toruń, Poland

**Keywords:** DNA methylation, environmental exposure, allergy, *IL4*, *IL5*, *IL10*, *IFNG*, *FOXP3*

## Abstract

**Objectives:**

Epigenetic dynamics has been indicated to play a role in allergy development. The environmental stimuli have been shown to influence the methylation processes. This study investigated the differences in CpGs methylation rate of immune-attached genes between healthy and allergic infants. The research was aimed at finding evidence for the impact of environmental factors on methylation-based regulation of immunological processes in early childhood.

**Methods:**

The analysis of methylation level of CpGs in the *IL4*, *IL5*, *IL10*, *IFNG* and *FOXP3* genes was performed using high resolution melt real time PCR technology. DNA was isolated from whole blood of Polish healthy and allergic infants, with food allergy and/or atopic dermatitis, aged under six months.

**Results:**

The significantly lower methylation level of *FOXP3* among allergic infants compared to healthy ones was reported. Additional differences in methylation rates were found, when combining with environmental factors. In different studied groups, negative correlations between age and the *IL10* and *FOXP3* methylation were detected, and positive - in the case of *IL4*. Among infants with different allergy symptoms, the decrease in methylation level of *IFNG*, *IL10*, *IL4* and *FOXP3* associated with passive smoke exposure was observed. Complications during pregnancy were linked to different pattern of the *IFNG*, *IL5*, *IL4* and *IL10* methylation depending on allergy status. The *IFNG* and *IL5* methylation rates were higher among exclusively breastfed infants with atopic dermatitis compared to the non-breastfed. A decrease in the *IFNG* methylation was noted among allergic patients fed exclusively with milk formula. In different study groups, a negative correlation between *IFNG*, *IL5* methylation and maternal BMI or *IL5* methylation and weight was noted. Some positive correlations between methylation rate of *IL10* and child’s weight were found. A higher methylation of *IL4* was positively correlated with the number of family members with allergy.

**Conclusion:**

The *FOXP3* methylation in allergic infants was lower than in the healthy ones. The methylation profile of *IL4*, *IL5*, *IL10*, *IFNG* and *FOXP3* associated with environmental exposures differed between the studied groups. The results offer insights into epigenetic regulation of immunological response in early childhood.

## 1 Introduction

Food allergy (FA) is a complex, multifactorial disease that is becoming a worldwide health problem. Its prevalence is increasing in both Western and developing countries affecting up to 10% of the populations, with the greatest incidence observed among younger children (1, 2). Cow’s milk and egg allergy are two of the most common food allergies in most countries (2) and most of them resolve within the first few years of life (3, 4).

In normal conditions, antigen-presenting immune cells including dendritic cells (5), macrophages (6), and T regulatory (Tregs) cells (7) inhibit an inappropriate immune response to food antigens, whereas in allergic individuals, it comes to dysregulation of normal immune tolerance and in consequence, to FA development (1). It was found that IgE-mediated food allergy results from a Th2 immune response of the adaptive immune system to specific food-derived antigens (8). By extension, Th1/Th2 lineage determination mediated by multitude of factors, including the cytokine environment, is critical to the development of food allergy (9). Th1 cells are mainly responsible for interferon gamma (IFNG) production and mounting a protective response against infectious agents. Interleukin 4 (IL4), interleukin 5 (IL5) and interleukin 10 (IL10) are secreted by Th2 cells, which are involved in allergic responses and humoral immunity (10). Any disruption of the cytokine secretion, especially increased IL4 and/or decreased IFNG, are considered to be a major factor contributing to allergy development (11). The IL5 contributes to the development of allergic diseases via a very complicated process of inflammation engaging the T cells and granulocytes (12). The IL10 is a regulator of the immune response and repressor of inflammation. A modulation of the Th1 and Th2 responses by T cell-derived IL10 may lead to the acquisition of immunotolerance (13). The expression pattern of transcription factor Forkhead Box Protein 3 (FOXP3) in Tregs was shown to be associated with the IgE-dependent food allergy and acquisition of tolerance in infants with cow’s milk allergy (14, 15).

It is suggested that FA mechanisms may involve the interactions between genetics, epigenetics and environmental factors. The epigenetic changes are believed to provide a possible account of the influence of ambient exposures on gene expression, dysregulating immune processes as a result (1, 9). The differences between methylation profiles of single-food and multi-food-allergic individuals were observed, suggesting that the epigenome changes play a significant role in differentiation of B cells in allergic processes (16). The potential of analysis of 96 CpG’s methylation status to predict a clinical response to food challenge in food-sensitized infants was demonstrated (17). It was found that DNA methylation status of Th1/Th2- related genes like *IL4*, *IL5*, *IL10* and *IFNG* was different between children with cow’s milk allergy and those tolerant to cow’s milk (18). Similarly, the association of the *FOXP3* gene methylation with milk’s allergy status in young allergic patients was observed (19). The study by Syed et al. (20) showed an *FOXP3* methylation decline throughout the peanut oral immunotherapy course.

There are few papers on the role of DNA methylation of Th1/Th2 loci in the food allergy/atopic dermatitis development in infants. The studies performed thus far focused preferentially on the dynamics of RNA and protein levels depending on allergy status. Besides, the studies were very small in terms of sample sizes. In the case of infants, the cord blood has often been used to infer differences between the healthy and allergic participants, whereas in older children or adults, the peripheral blood mononuclear cells (PBMC), single blood cells lineages or purified T-cell populations were examined. It cannot be ruled out that cord blood might contain contamination with maternal cells. In turn, PBMC’s collection requires large amounts of blood to be drawn, which can be difficult in the case of infants.

The first objective of the current investigation was to examine the methylation level of CpG’s spanning promoter sequences of the *IL4*, *IL5*, *IL10* and *IFNG* genes and Treg-specific demethylated region (TSDR) within the *FOXP3* gene in whole blood collected from infants. The supplementary goal was to check if a few drops of blood deposited on the FTA card would be sufficient for testing, which would be extremely beneficial in the case of the youngest patients. For the purpose of determining methylation status, a very simple and cost-effective real-time PCR technology was employed. The next goal was to detect any differences in methylation of selected loci between infants with and without allergy, in order to infer whether the TH1/TH2 methylation profile from whole blood may be predictive in allergy development. Additionally, the study was aimed at finding evidence for the impact of environmental factors on methylation dynamics in allergic and healthy groups that would help in better understanding of molecular basis of allergy.

## 2 Materials and methods

### 2.1 Subject recruitment

As patients, we enrolled infants with atopic dermatitis (AD) and/or food allergy (FA) under six months of age that were hospitalized in the Department of Pediatrics, Allergology and Gastroenterology, Collegium Medicum, Nicolaus Copernicus University, Poland and from Gastrological and Allergological Outpatient Clinics in Bydgoszcz, Poland. As controls, during the same study period, healthy infants without symptoms indicating allergy visiting the medical centre were also recruited. The inclusion and exclusion criteria as well as medical data were described in detail previously (21).

Finally, after rejection of participants who did not meet all the requirements, the study sample consisted of 89 controls and 138 patients with allergy suspicion (for simplicity, this group was further referred to as allergic patients) including 15, 38 and 85 with atopic dermatitis (AD), food allergy (FA) or atopic dermatitis/food allergy (ADFA), respectively. For some analyses infants with ADFA were coupled with those with FA or AD (FA+ADFA group and AD+ADFA group, respectively).

### 2.2 Sample collection

Peripheral blood samples from patients and healthy subjects were collected on FTA cards during medical appointments and delivered in short order to the laboratory for further processing.

### 2.3 Methylation analysis

We investigated the methylation profiles of selected CpG’s sites located in *IL4* (Chr5:132673908, Chr5:132673939, Chr5:132673992, Chr5:132674040), *IL5* (Chr5:132556873, Chr5:132556876, Chr5:132556881, Chr5:132556887, Chr5:132556894, Chr5:132556902, Chr5:132556906, Chr5:132556911, Chr5:132556913, Chr5:132556929, Chr5:132556932, Chr5:132556947, Chr5:132556950, Chr5:132556956, Chr5:132556958, Chr5:132556962, Chr5:132556978), *IL10* (Chr1:206772789, Chr1:206772819, Chr1:206772821, Chr1:206772842), *IFNG* (Chr12:68159573, Chr12:68159617, Chr12:68159623) gene promoters and in the TSDR of *FOXP3* gene (ChrX:49260767, ChrX:49260786, ChrX:49260795, ChrX:49260803, ChrX:49260807, ChrX:49260813, ChrX:49260816, ChrX:49260826, ChrX:49260834).

DNA from whole blood deposited on FTA card was extracted by GeneMATRIX Bio-Trace DNA Purification Kit (EurX) according to the manufacturer’s instructions. DNA concentration was estimated with Quantifiler Duo Quantification kit (Thermo Fisher Scientific). 500 ng of DNA was bisulfite-converted using the EpiTect Fast DNA Bisulfite Kit (Qiagen) following the manufacturer’s protocol. PCR was performed in a 10 μL reaction volume. 10 ng of converted DNA were added to each well which contained 1 × HRM Master Mix^®^ (Thermo Fisher Scientific) and 0.2 μM of each primer (for details see **Supplementary Table S1**). The amplification consisted of 10 min at 95°C and 40 cycles of the following steps: denaturation 95°C, 15 s and annealing/extension 61°C (exception: 60°C in case of *IL4* reaction), 1 min. High resolution melting analyses were performed at the temperature ramping and fluorescence acquisition setting recommended by the manufacturer. Real Time PCR amplification of the DNA (for quantification purposes as well as methylation analysis) was carried out using ViiA7 apparatus (Thermo Fisher Scientific) equipped with the Ruo software (Version 1.1). The EpiTect PCR Control DNA Set consisting of Human Methylated and Non-Methylated DNA standards were purchased from Qiagen, and mixed to receive the standard curve containing 0, 10, 25, 50, 75 and 100% of methylated template. The methylation level of each test sample was determined by interpolation of the data generated from the linear regression analysis of the standard curve as described in (22). Additionally, the methylation analysis products were verified by direct sequencing using Sanger method.

### 2.4 Statistical analysis

The normality of data was checked with the Shapiro–Wilk test and homogeneity of variance by Levene’s test. Clinical categorical data were analysed using two-tailed Fisher’s exact and/or Chi-square tests. The Kruskal–Wallis one way ANOVA test was used to evaluate the differences among continuous variables. Bonferroni correction for multiple comparisons was applied. Correlations between demographic/clinical variables and methylation levels in different compartments were tested using Spearman’s rho. For all statistical tests a significance level of 0.05 (p) was used. All analyses were conducted by SPSS for Windows (PS IMAGO PRO 8.0).

## 3 Results

The characteristics of the study and control groups are given in **Table 1**. Generally, the study and control cohorts are similar, but some significant differences were observed, including mother’s weight/BMI (which is higher in the control group) and familial history of allergy. The significant findings described in sections below were shown in figures (numbered 1-10). All results of statistical analyses were presented in **Supplementary Tables S2–S13)**.

**Table 1:** The characteristics of the study and control group.

Variables		Control group n = 89	Allergic group n = 138	FA group n = 38	ADFA group n = 85	AD group n = 15
**Child’s age (weeks), mean ± SD**		14.87 ± 6.62	16.63 ± 6.89	16.13 ± 6.65	16.58 ± 7.01	18.20 ± 7.01
**Sex, n (%)**	**Females**	46 (51.7)	57 (41.3)	17 (44.7)	34 (40.0)	6 (40)
	**Males**	43 (48.3)	81 (58.7)	21 (55.3)	51 (60.0)	9 (60)
**Child’s birth weight (kg), mean ± SD**		3.36 ± 0.59	3.47 ± 0.46	3.36 ± 0.53	3.50 ± 0.43	3.59 ± 0.37
**Child’s current weight (kg), mean ± SD**		7.09 ± 1.37	7.18 ± 1.33	6.09 ± 1.35	6.42 ± 1.36	6.96 ± 1.58
**Exclusive breastfeeding, n (%)**		38 (42.7)	57 (41.3)	15 (39.5)	36 (42.3)	6 (40.0)
**Exclusive milk formula feeding, n (%)**		42 (47.1)	67 (48.5)	20 (52.6)	39 (45.9)	8 (53.3)
**Place of residence, n (%)**	**village**	9 (10.1)	7 (5.1)	2 (5.3)	4 (4.7)	2 (5.3)
	**suburbs**	14 (15.7)	36 (26.1)	12 (31.6)	20 (23.5)	12 (31.6)
	**city**	66 (74.2)	95 (68.8)	24 (63.2)	61 (71.8)	24 (63.2)
**Mother’s age (years), mean ± SD**		29.37 ± 5.11	30.04 ± 4.89	29.50 ± 5.01	30.71 ± 4.65	27.67 ± 5.33
**Father’s age (years), mean ± SD**		31.96 ± 5.08	32.35 ± 5.10	31.74 ± 5.36	32.96 ± 4.84	30.40 ± 5.59
Mother’s weight (kg), mean ± SD *		65.35 ± 12.21	62.20 ± 10.17	65 ± 9.16	61.70 ± 10.72	58.15 ± 7.47
Mother’s BMI, mean ± SD **		23.71 ± 4.12	22.52 ± 3.41	23.49 ± 3.24	22.25 ± 3.53	21.76 ± 2.76
**Siblings, n (%)**		35 (39.3)	59 (42.8)	16 (42.1)	37 (43.5)	6 (40)
**Animals at home, n (%)**		33 (37.1)	60 (43.5)	20 (52.6)	32 (37.6)	8 (53.3)
**Passive smoking exposure, n (%)**	**During pregnancy**	17 (19.1)	29 (21)	9 (23.7)	16 (18.8)	4 (26.7)
	**After birth**	25 (28.1)	35 (25.4)	9 (23.7)	19 (22.4)	7 (46.7)
**Pregnancy complications, n (%)**		33 (37.1)	45 (32.6)	14 (36.8)	24 (28.2)	7 (46.7)
Number of family members with allergy, n (%) **	**0**	46 (51.7)	56 (40.6)	18 (47.4)	26 (30.6)	12 (80)
	**1**	32 (36)	40 (29)	12 (31.6)	27 (31.8)	1 (6.7)
	**2**	11 (12.4)	33 (23.9)	7 (18.4)	25 (29.4)	1 (6.7)
	**3**	0 (0)	9 (6.5)	1 (2.6)	7 (8.2)	1 (6.7)

* significant p value for comparisons between control and allergic groups.

** significant p value for comparisons between control and allergic groups/FA, AD and ADFA groups.

### 3.1 Methylation status, presence of allergy and the type of symptoms

**Figure 1:**
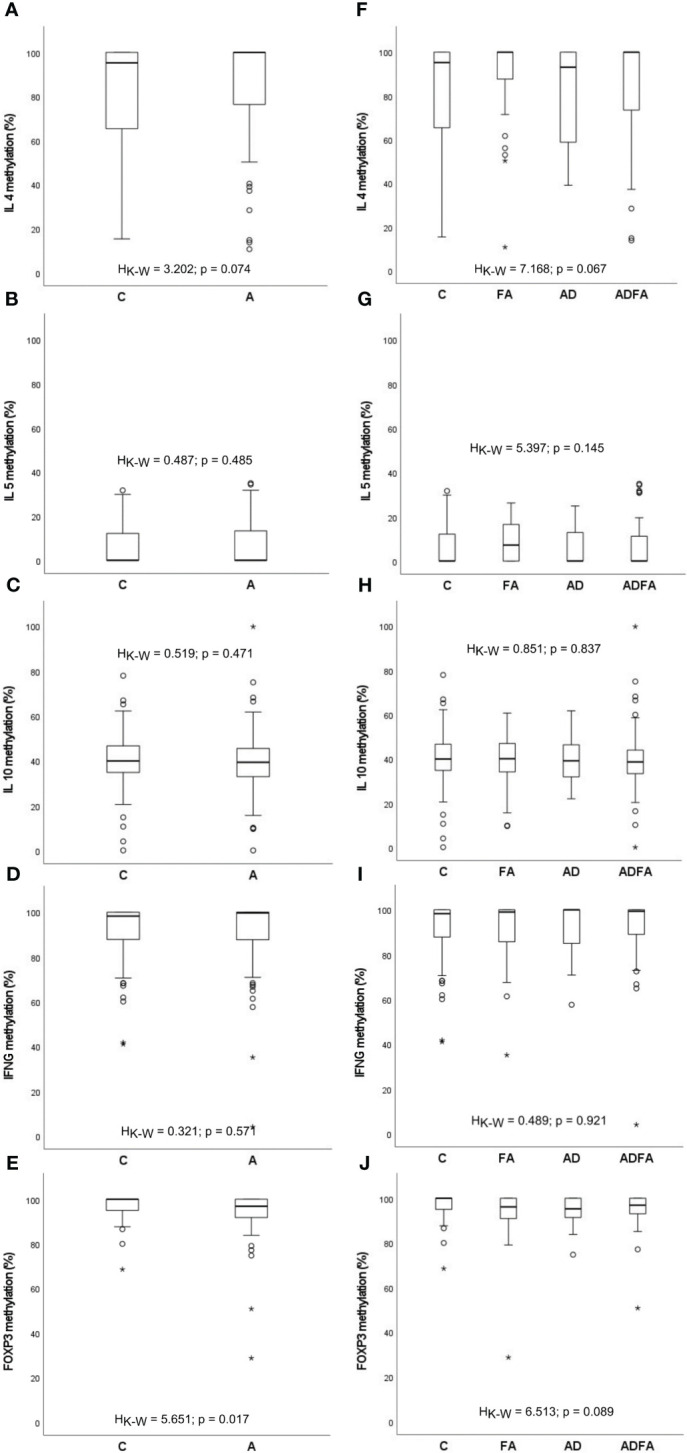
DNA methylation rate of the *IL4*, *IL5*, *IL10*, *IFNG* and *FOXP3* genes observed in the study population. **(A-E)** comparisons between control and allergic groups, **(F-J)** comparisons between control group and groups with FA, AD and ADFA. °C and * – the outliers, H_K-W_ – Kruskal-Wallis ANOVA coefficient, level of significance p<0.05.

The present study concentrates on methylation profiles of gene promoters of the immune response-attached factors IL4, IL5, IL10, IFNG and TSDR of FOXP3. The assessments of the methylation level in particular loci were based on relevant standard curves. A similar distribution of methylation for allergic and healthy donors was observed. Methylation interquartile values were fluctuated depending on locus: for *IFNG* ranging from approximately 82% to 100%, for *FOXP3* from 90% to 100%, for *IL4* from 56% to 100%, for *IL10* from 31% to 47% and for *IL5* from 0% to 16%. In the case of the first three loci, most values were between 95% and 100%, whereas the *IL5* methylation rate mostly assumed a value near 0%. Some outstanding values for all loci were also observed. The methylation rates of the *IL4*, *IL5*, *IL10*, *IFNG* and *FOXP3* genes are presented in **Figure 1**. No significant differences were found between controls and all allergic patients with the exception of the *FOXP3* gene for which lower percentage of methylation in allergic infants was observed (H_K-W_=5.651; p=0.017). On the other hand, the distributions of the methylation level for all genes did not differ between healthy participants and those with FA, AD and ADFA, analysed separately.

### 3.2 The impact of environmental exposures on methylation dynamics in the *IL4*, *IL5*, *IL10*, *IFNG* and *FOXP3* genes

#### 3.2.1 Demographic/social data

Comparison of the percentage of methylation between the healthy and allergic groups (analysed separately or together) in the context of gender did not exhibit any significant distinctions. No statistically significant differences in methylation levels of the genes between any studied groups were found, regardless of having siblings or animals. Similarly, no connection between the methylation status and place of residence (village, suburbs or city) was detected.

**Figure 2:**
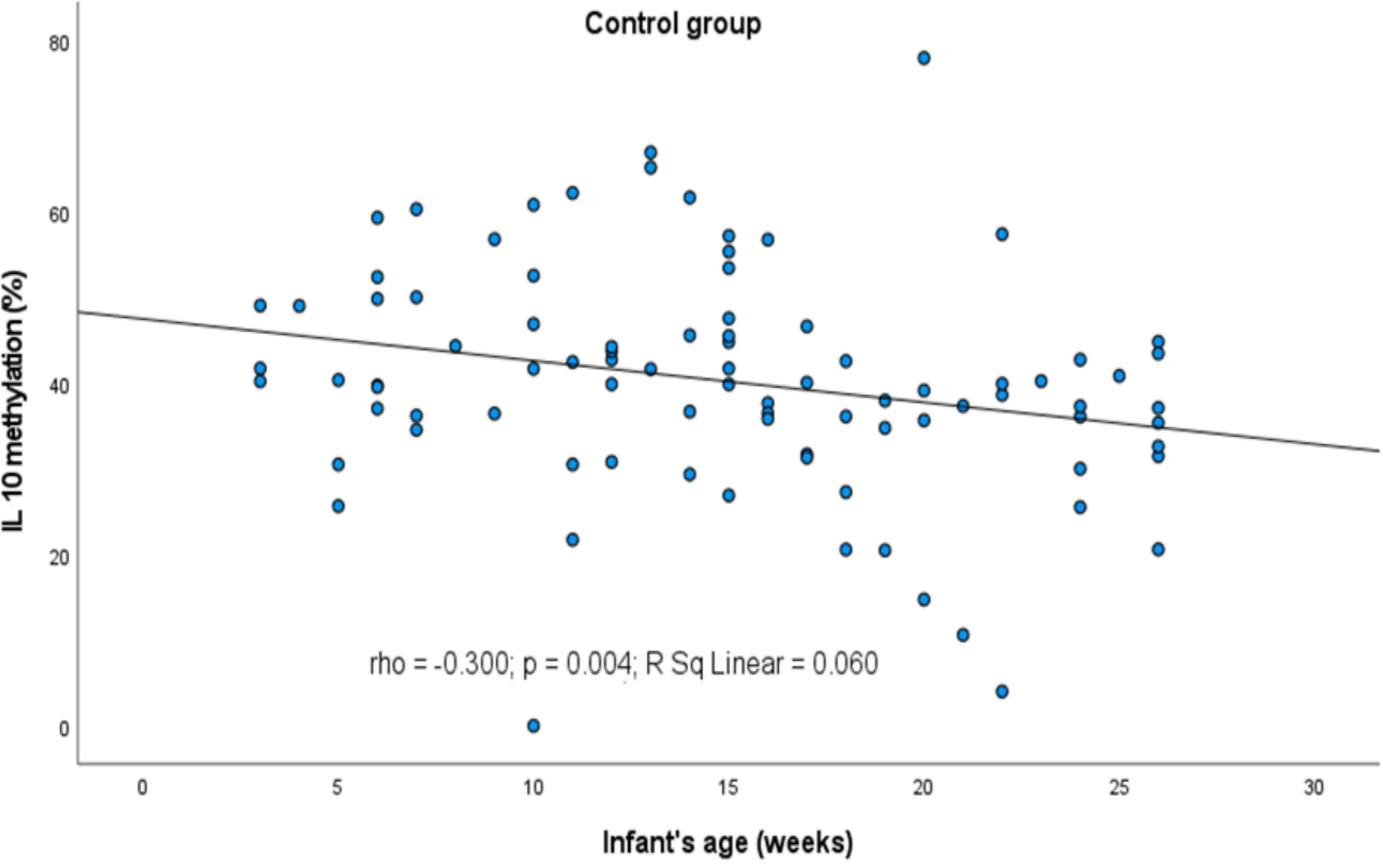
Significant correlation between DNA methylation level of *IL10* promoter and infant’s age. rho – Spearmans’ rho coefficient, level of significance p<0.05.

**Figure 3:**
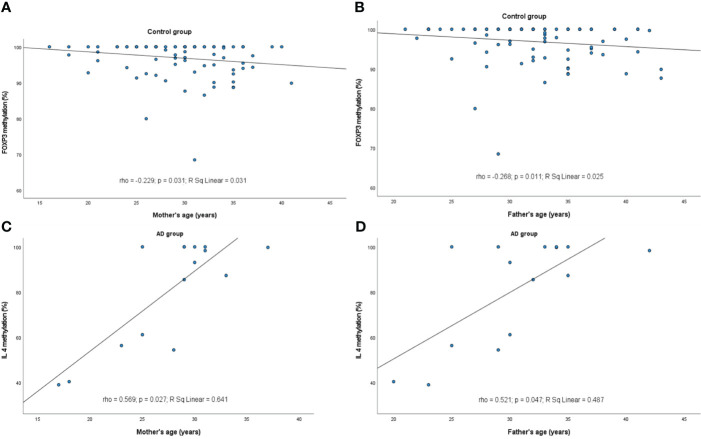
Significant correlations between DNA methylation level and parent’s age: **(A)** *FOXP3* methylation rate in control group and maternal age, **(B)** *FOXP3* in control group and paternal age, **(C)** *IL4* in AD group and maternal age, **(D)** *IL4* in AD group and paternal age. rho – Spearmans’ rho coefficient, level of significance p<0.05.

However, some relations between the infants’ and parents’ age and the methylation profile of the genes were observed (**Figures 2**, **3**, respectively). It turned out that the older the child, the lower was the *IL10* methylation in the control group (rho=-0.300, p=0.004). Besides, the older the mother or father, the lower was the *FOXP3* methylation in controls (rho=-0.229, p=0.031 and rho=-0.268, p=0.011, respectively). In turn, the opposite relationship between the *IL4* methylation and age of mother and father in the AD group was shown (rho=0.569, p=0.027 and rho=0.521, p=0.047, respectively).

#### 3.2.2 Cigarette smoking

**Figure 4:**
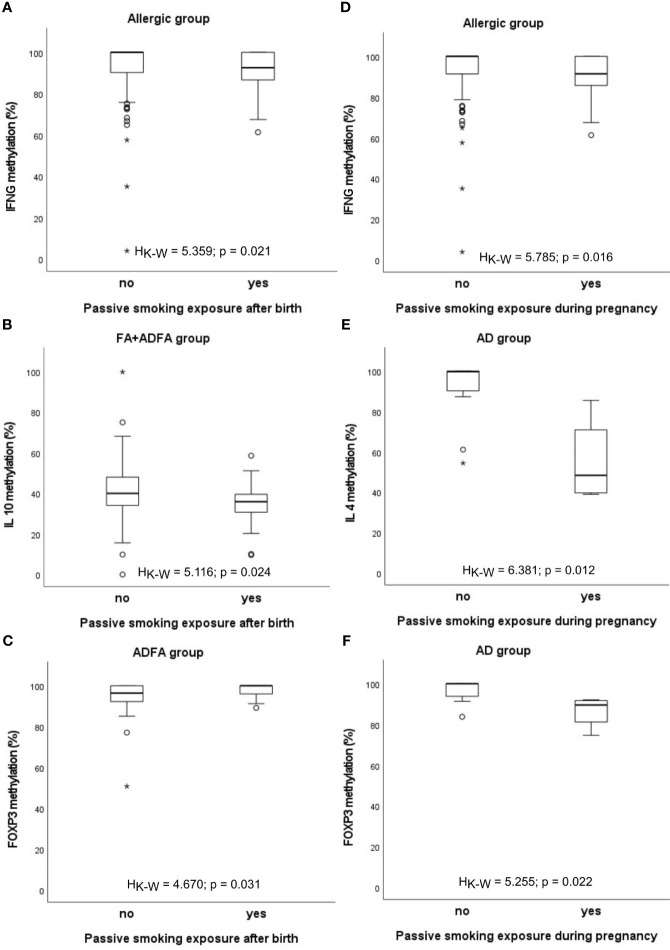
Significant associations between DNA methylation level and infant’s passive smoking exposure: **(A-C)** after birth, **(D-F)** during pregnancy. °C and * – the outliers, H_K-W_ – Kruskal-Wallis ANOVA coefficient, level of significance p<0.05.

When cigarette smoking exposure is taken into account, some differences in the methylation levels appeared in a manner that in infants subjected to second-hand smoke the methylation level has mainly declined (**Figure 4**). In details, the *IFNG* methylation level decrease was observed in participants with any allergy (H_K-W_=5.359, p=0.021), the *IL10* methylation level was lower among FA+ADFA patients (H_K-W_=5.116, p=0.024), whereas the *FOXP3* methylation level was higher among those with ADFA (H_K-W_=4.670, p=0.031), while smoking exposure was taking place after the birth of an infant.

The same relationship was found between the percentage of methylation and smoking exposure during pregnancy. The methylation rate of *IFNG* promoter was lower in allergic children and in the group consisting of FA+ADFA donors (H_K-W_=5.785, p=0.016 and H_K-W_=4.130, p=0.042, respectively). Similarly, methylation level of *IL4* and *FOXP3* genes diminished among children with AD (H_K-W_=6.381, p=0.012 and H_K-W_=5.255, p=0.022, respectively).

#### 3.2.3 Complications during pregnancy

Abnormal course of pregnancy was shown to be associated with changes of methylation degree. Different abnormalities were diagnosed in mothers including gestational diabetes, hypothyroidism, hypertension, anaemia, oligoamnios, hydramnion, bacterial infections, mycoses and others. One of the most frequently diagnosed complications of pregnancy in the study was gestational diabetes but few cases were limited only to this disease. The presence of several comorbidities was prevalent so it was decided to carry out statistical analyzes for pregnancy complications in general, without separating them into individual disease entities.

**Figure 5:**
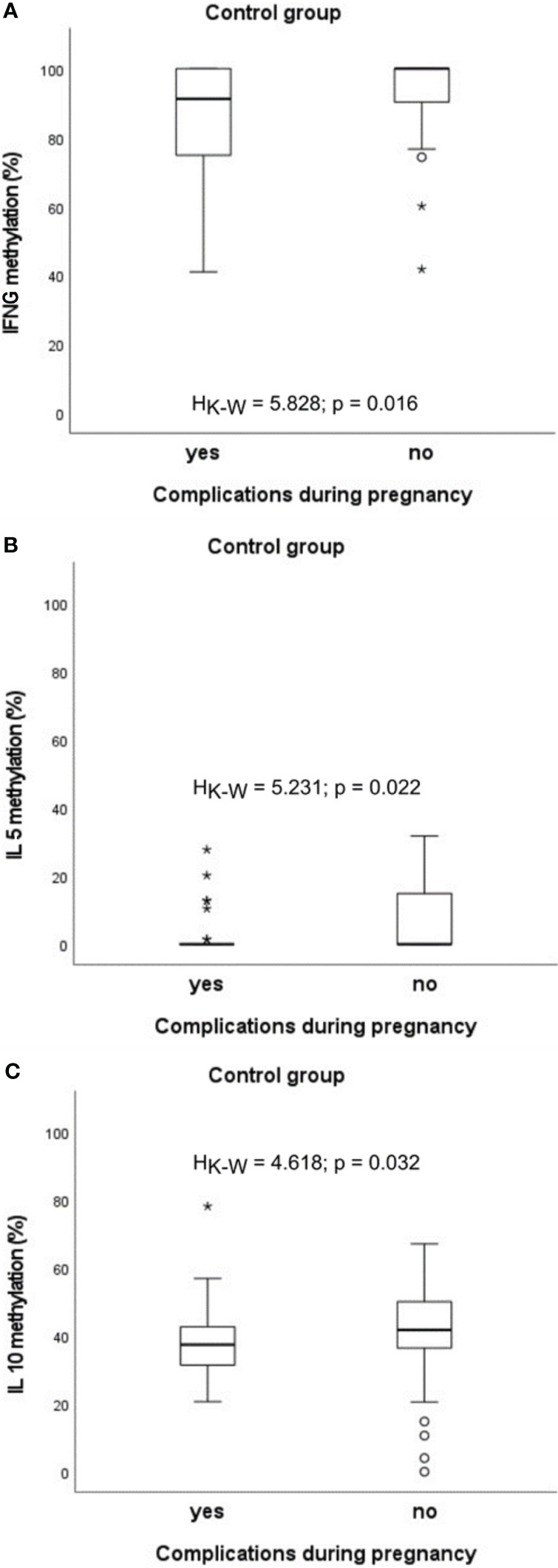
Significant associations between DNA methylation level and the presence of complications during pregnancy in the control group: **(A)** in case of *IFNG*, **(B)** in case of *IL5*, **(C)** in case of *IL10*. °C and * – the outliers, H_K-W_ – Kruskal-Wallis ANOVA coefficient, level of significance p<0.05.

In the healthy group, significantly lower methylation of *IFNG* (H_K-W_=5.828, p=0.016), *IL5* (H_K-W_=5.231, p=0.022) and *IL10* (H_K-W_=4.618, p=0.032) promoters was found (**Figure 5**).

**Figure 6:**
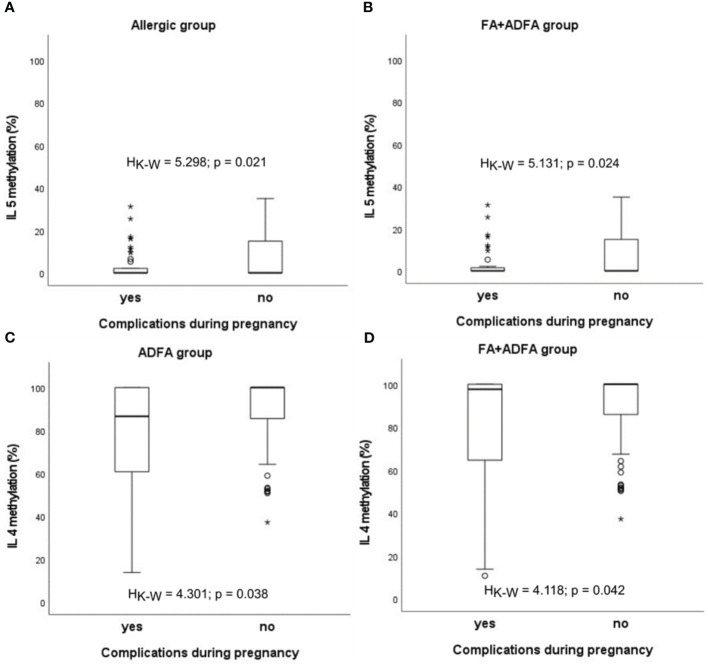
Significant associations between DNA methylation level and the presence of complications during pregnancy in patients: **(A, B)** in case of *IL5*, **(C, D)** in case of *IL4*. °C and * – the outliers, H_K-W_ – Kruskal-Wallis ANOVA coefficient, level of significance p<0.05.

Similar statistically significant relations between an abnormal pregnancy and methylation status were observed for *IL5* in infants with allergy (H_K-W_=5.298, p=0.021) and in those from the FA+ADFA group (H_K-W_=5.131, p=0.024), whereas for *IL4* in patients with ADFA (H_K-W_=4.301, p=0.038) and among the FA+ADFA infants (H_K-W_=4.118, p=0.042) (**Figure 6**).

#### 3.2.4 The type of infant feeding

**Figure 7:**
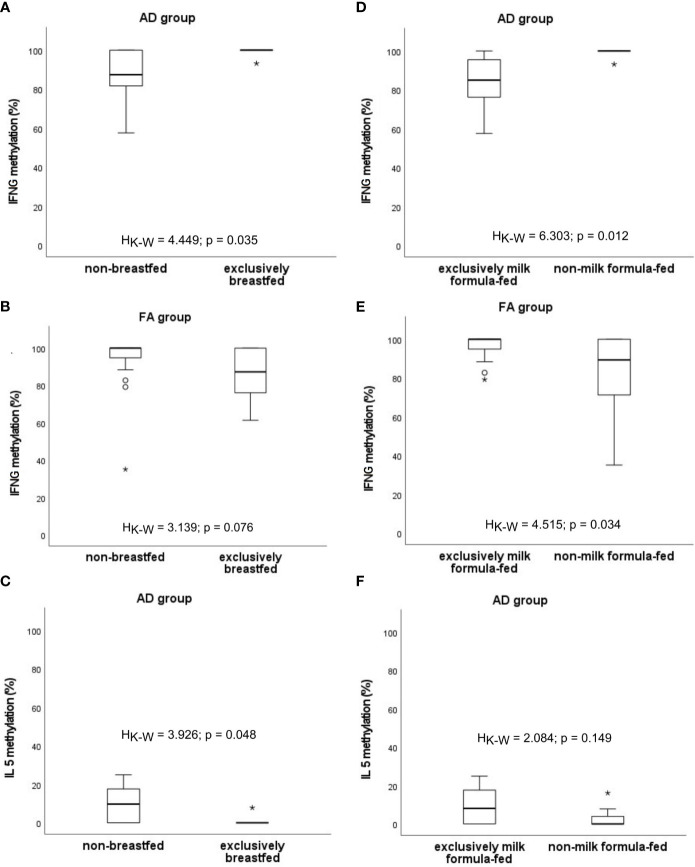
Significant associations between DNA methylation level and the type of infant feeding: **(A–C)** excusively breastfed vs. non-breastfed, **(D–F)** excusively milk formula-fed vs. non-milk formula-fed. °C and * – the outliers, H_K-W_ – Kruskal-Wallis ANOVA coefficient, level of significance p<0.05.

The possible impact of the type of feeding on the level of methylation was analysed (**Figure 7**). It was shown that percentage of methylation of *IFNG* is higher among exclusively breastfed infants with AD compared to those who were not breastfed at all (H_K-W_=4.449, p=0.035) and lower among exclusively milk formula-fed compared to those who were never fed by milk formula (H_K-W_=6.303, p=0.012). The opposite observation in patients with FA was found (H_K-W_=3.139, p=0.076; H_K-W_=4.515, p=0.034, respectively), whereby results for exclusively breastfed infants were not significant. Additionally, the significantly decreased methylation of *IL5* among infants with AD who were exclusively breastfed, was observed (H_K-W_=3.926, p=0.048). A similar, but not significant trend was noted for milk formula feeding (H_K-W_=2.084, p=0.149).

#### 3.2.5 BMI/weight of mother or infant

**Figure 8:**
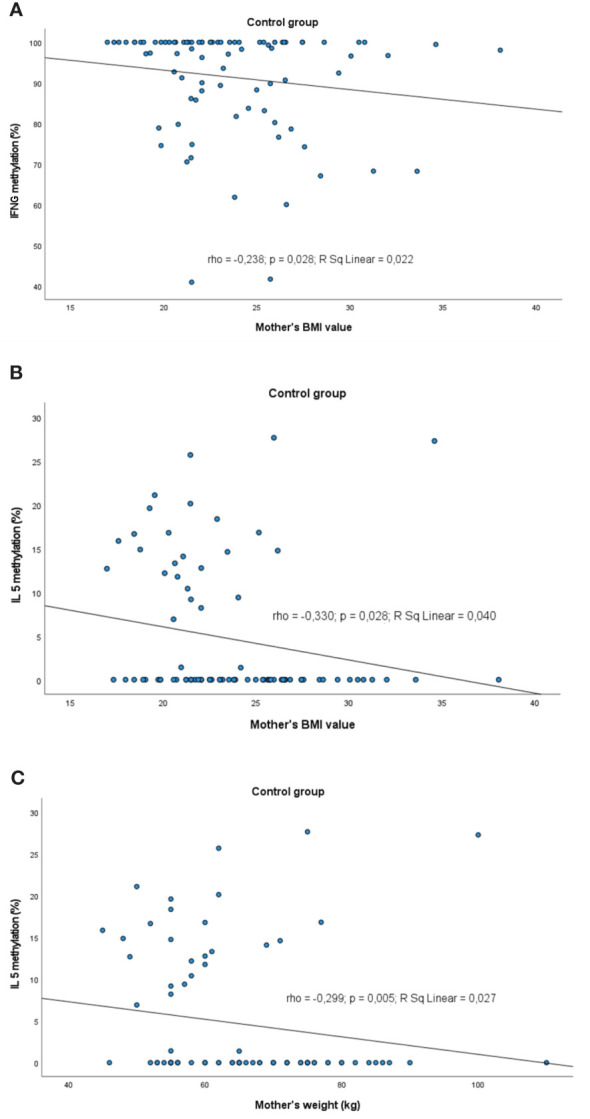
Significant correlations between DNA methylation level and maternal weight/BMI: **(A)** correlation between *IFNG* and maternal BMI in control group, **(B)** correlation between *IL5* methylation and maternal BMI in control group, **(C)** correlation between *IL5* methylation and maternal weight in control group. rho – Spearmans’ rho coefficient, level of significance p<0.05.

**Figure 9:**
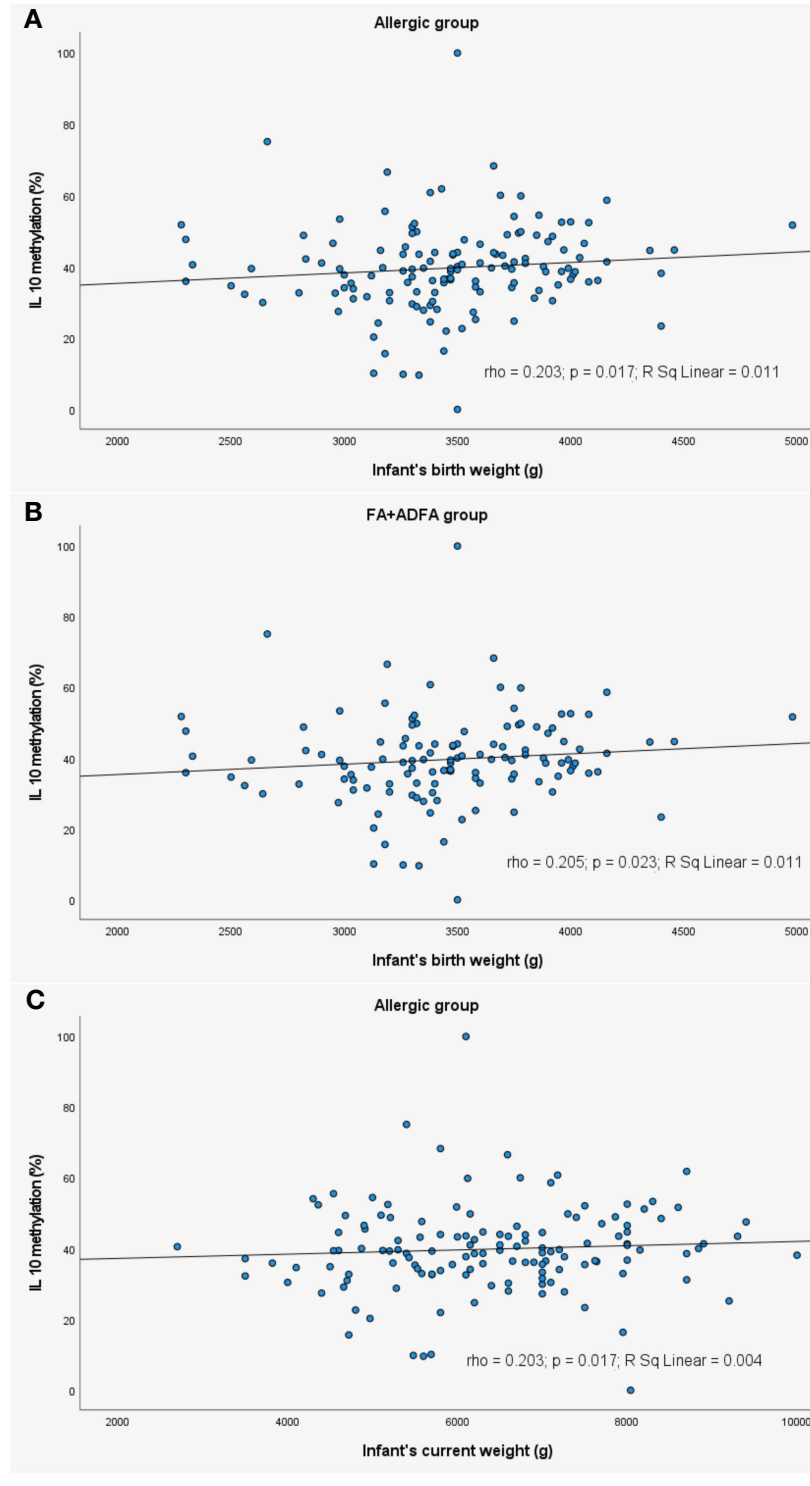
Significant correlations between DNA methylation level and infant’s weight: **(A)** correlation between *IL10* methylation and infant’s birth weight in allergic group, **(B)** correlation between *IL10* methylation and infant’s birth weight in FA+ADFA group, **(C)** correlation between *IL10* methylation and infant’s current weight in allergic group. rho – Spearmans’ rho coefficient, level of significance p<0.05.

Analyses of correlation between methylation profiles and values of maternal weight/BMI are presented in **Figure 8** and child’s weight in **Figure 9** as well.

A slight negative correlation between the mother BMI and methylation profile was observed. The higher the BMI value, the lower was the methylation level of: *IFNG* (rho=-0.238, p=0.028) and *IL5* (rho=-0.330, p=0.028) in controls. The same trend was observed for the *IL5* methylation status in controls in the case of the mother’s weight (rho=-0.299, p=0.005).

In the allergic and FA+ADFA groups, the increase in the level of the *IL10* methylation was observed with an increase in the birth weight of the child (rho=0.203, p=0.017 and rho=0.205, p=0.023, respectively).

The current child’s weight was positively correlated with *IL10* methylation level in patients with allergy (rho=0.203, p=0.017).

#### 3.2.6 History of familial allergy

**Figure 10:**
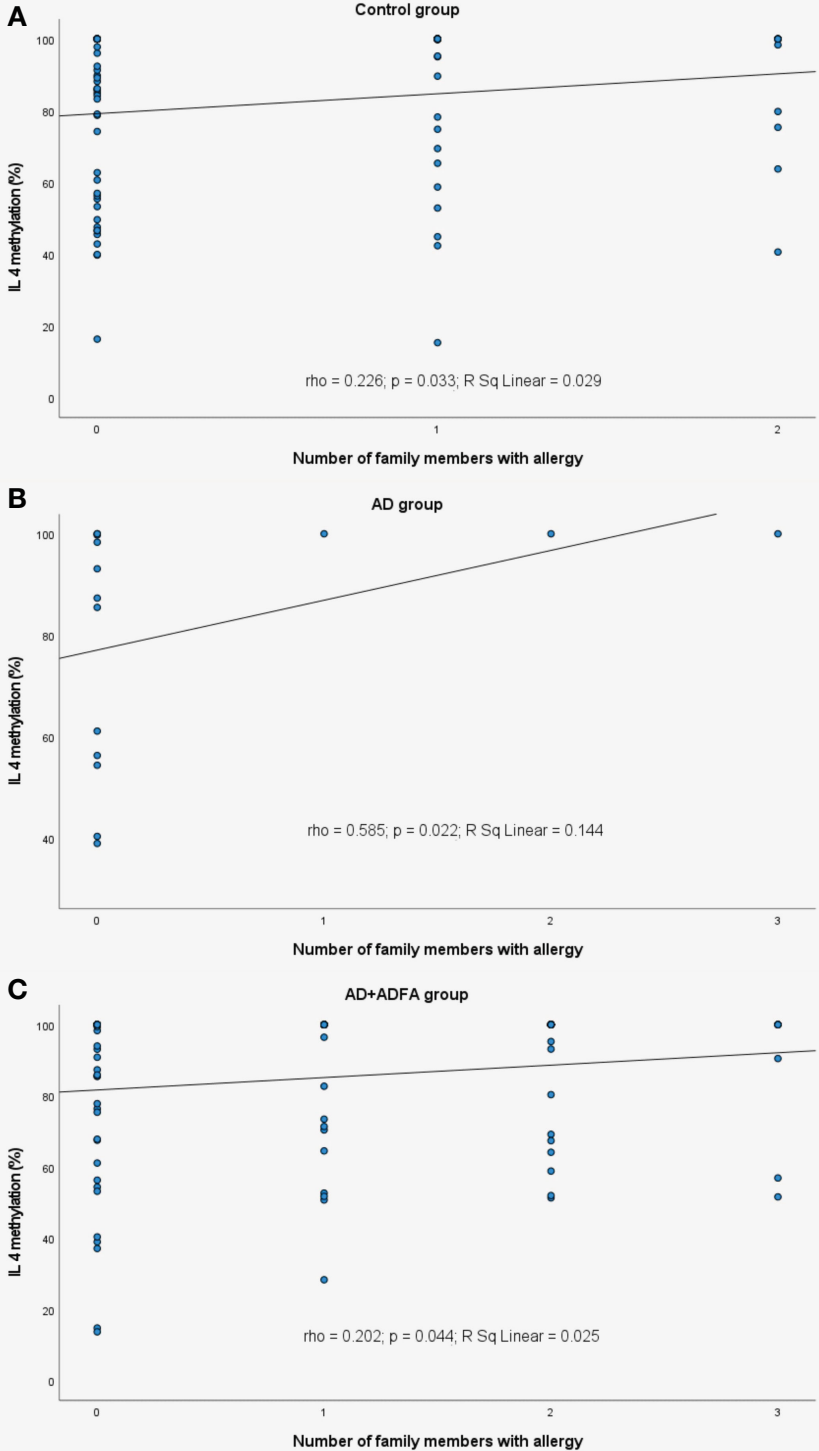
Significant correlations between DNA methylation level of *IL4* and the number of family allergic members: **(A)** in control group, **(B)** in AD group, **(C)** in AD+ADFA group. rho – Spearmans’ rho coefficient, level of significance p<0.05.

A weak positive correlation between the number of allergic family members and the *IL4* methylation was observed in the healthy subjects (rho=0.226, p=0.033), AD patients (rho=0.585, p=0.022) and AD+ADFA patients (rho=0.202, p=0.044) (**Figure 10**).

## 4 Discussion

### 4.1 Methylation status, presence of allergy and the type of symptoms

Previous studies have shown that epigenetic changes, especially the pattern of DNA methylation, were associated with food allergy and other allergic diseases, as reviewed in (23). Methylation in CpG islands within gene promoters is believed to silence gene expression. Also, a strong correlation with gene expression has been shown for DNA methylation in regions located even up to 2 kb from known CpG islands (24).

All researches the papers of which are cited in the current work examined fresh blood samples but we used blood spotted on FTA cards. Since DNA extraction and even transport to the place of proper storage cannot always be performed at the time of sample collection (i.e. immediately after the visit), whole blood samples must be stored in conditions preventing the decline of the yield and quality of DNA, including DNA methylation. FTA classic cards are commonly used for blood, saliva and sperm transport and storage providing reliable results of classic DNA analysis like genotyping or sequencing. More recently, their usefulness was confirmed for epigenetic analysis (25–27).

Since different methylation profiles may act as potential biomarkers of modifiable disease pathways, the methylation profiles of selected CpG’s sites spanning regulatory regions of the *IL4*, *IL5*, *IL10*, *IFNG* and *FOXP3* genes from infants with FA, AD and with symptoms of both (ADFA), as well as healthy subjects were investigated. To simplify the workflow as much as possible, the MS-HRM methodology was used that enables assessment of the overall methylation percentage of the entire amplicon in a particular sample by comparison with melting standard curves created by different dilution ratios of methylated and unmethylated control DNAs. Using this method, even small differences (5%–10%) in DNA methylation could be detected, allowing to assess the contribution of methylated DNA from subpopulations of cells within heterogeneous populations (28).

Comparing the distribution of methylation of the TH1/TH2 cytokine and *FOXP3* genes from patients with those from the control group demonstrated a significant difference for the *FOXP3* gene only. It was shown that lower percentage of methylation occurred in allergic infants. But measuring the methylation level in the CpG loci of healthy infants up against the ones with FA, AD or ADFA, respectively, did not differ significantly. In other studies methylation of the same loci had the values ranging from approximately 35% to 85% for *IL4*, *IL5* and *IFNG* (18) and from 75% to 100% for *FOXP3* (19) depending on the allergy status. It has been proved that DNA methylation profiles of CpGs in the promoter region of *IL4*, *IL5*, *IL10* and *IFNG* (18) as well as the demethylation status of TSDR in *FOXP3* (19) clearly separated patients with cow’s milk allergy from controls and children who outgrew CMA. The *IL4* and *IL5* DNA methylation was significantly lower, and the *IL10*, *IFNG* and *FOXP3* DNA methylation was higher in active IgE-mediated CMA patients compared to the other two groups. The discrepancy between methylation rates obtained in this and other studies (18, 19) can result from distinct material used for examination, since we used whole blood samples, whereas other groups tested peripheral blood mononuclear cells (PBMCs) isolated from whole blood samples. Blood is a complex mixture of many different specialized cell types with varying methylation profiles (29). It can influence detected methylation level and, for example, may have a dilution effect. For example, normal proportion of T cells equals 20% of white blood cells (29), so analysing PBMC will give distinct methylation values for TH1/TH2-derived loci in comparison to whole blood. On the other hand, Yu et al. (30) identified relative hypermethylated states in gene promoters of neonatal CD4+ T cells, compared to those of adults. Others reported that neonatal CD4/CD45RO- T cells, but not CD8+ T cells, are hypermethylated at CpG within and adjacent to the *IFNG* promoter (31, 32). There is the opposite pattern of CpGs in both the promoter and the transcribed region in NK and B cells (hypo and hypermethylated, respectively) (33). The methylation differences spanning CpGs within and adjacent to the *IFNG* promoter were also noticed between neonatal CD4/CD45RO- T cells and CD8+ T cells and NK cells. The former were hypermethylated, whereas the latter were less methylated and were comparable with those of adults (31, 32). Hypermethylation of *IFNG* promoter down-regulated gene expression in neonatal T-cells, but for *IL4* a converse correlation was found in neonatal CD4+ T cells, compared to those of adults (30). It is possible that the pattern similar to adults occurs also in older children. It is worth mentioning that in the case of *IL4*, we reported a very wide range of methylation rate suggesting the methylation in this locus changes in a more individual manner. In turn, the level of methylation obtained in this study for *IL10* in whole blood fluctuated mainly between 31% and 47% but did not differ regardless of the presence of allergies. A similar range of values was shown for healthy controls and children who outgrew CMA, although the two groups could be distinguished from each other (18). In the case of allergic donors, a higher level of methylation has been shown (55-85%) (18). Cytokine IL10 is mainly expressed by monocytes, Th2 and regulatory T cells which have an inhibitory effect over the expression of Th1 cytokines (34). There are contradictory results on the presence of correlation between methylation of *IL10* promoter and the level of cytokine expression. The discrepancies concerned both the presence or absence of correlation, but also different CpG dinucleotides in the promoter of the *IL10* gene and different regulatory regions (promoter/intron) subjected to methylation (18, 35–39). Zheng et al. (40) have described in detail the intricate networks of relationships surrounding the dynamics of epigenetic changes associated with IL10. Moreover, IL10 can cause various effects depending on timing, dose, and location of expression; in some cases, the expected immunosuppressive activities are observed, while in others IL10 enhances immune or inflammatory responses (41). Coming back to our *FOXP3* methylation picture, lower methylation levels were observed among infants with allergies, in contrast to the results of others (19). We tested the blood of infants with active allergic symptoms at the time of inclusion. Some of these children developed tolerance at a later stage, and in some of them the initial diagnosis was verified (data not shown). The research of Paparo et al. (19) encompassed infants with CMA at diagnosis aged 5.5 months, the subject’s outgrown CMA and healthy participants aged 16.9 and 9 months, respectively. The same research group was examined by Canani et al. (18). Therefore, it is possible that differences in the methylation rates of *FOXP3* and TH1/TH2 cytokine genes, at least partially, result from distinct age of donors. In addition, it was pointed that the role of FOXP3 expression in unstimulated and milk-allergen stimulated different T-cell populations could not be the same and has to be elucidated (42). Another explanation for the lower *FOXP3* methylation could be that in the developing immune system, the inflammatory processes associated with the development of allergies start to arouse an earlier response, whereas nothing is happening in healthy children. All in all, these mechanisms are still unknown.

The above facts can explain the differences in the percentage of methylation between experiments. One could note it may be difficult to achieve sufficient signal strength of methylation changes in minor fraction, for example, Treg cells within the total white cell compartment. On the other hand, a diagnostic test that requires isolation of specific cell populations would be of far less utility in comparison to the one demanding only a small amount of unsorted whole blood, such as from finger prick. We have made such an attempt in this study. The lack of differences in our study may result from a dilution effect as mentioned above, but not only. It should be noted that in the case of *FOXP3* only, we certainely tested the same CpG’s set as elswere (19) because we used the same primers for amplification. For the remaining loci, we can confirm that we tested a similar region, the promoters, but are not sure if an identical set of CpG’s. It is documented that not all CpG’s in regulatory regions have the same methylation level in different donors [i.e. neonatal and adult T-cells differ in *IFNG* gene promoter particular CpG’s methylation pattern (32)], or in normal and affected conditions [i.e. the upstream CpGs at -408, -387, -385, and -355 bp had similar hypermethylated status in both the patients with rheumatoid arthritis and healthy donors, whereas the proximal CpG at -145 was hypomethylated to a much greater extent in the patients than in the controls (37)]. Another issue mentioned above concerns the age of the surveyed children, which varies in different publications. It has been found that methylation profiles of many loci are changing with age. Mulder et al. (43) characterized the genome-wide DNA methylation directions across age period spanning multiple time-points from birth to late adolescence. They have found that sites with decreasing levels of methylation around the age of 6 years were functionally enriched for immune-developmental pathways, whereas sites with increasing levels of methylation for the neurodevelopmental ones. It turned out that 11% of CpGs were undergoing methylation changes in a non-linear manner, mostly involving changes from birth to the age of 6 years, after which DNA methylation status was more stable. One should not forget about the differences in methylation patterns depending on the population origin, resulting from epigenome-wide studies in different populations, including Europeans, Africans, Latin Americans or Arabs. Additionally, health disparities between human populations may be partially elucidated by methylation differences (44). It was proposed that differences in the development of specific T cell memory to food and inhalant allergens during the first 2 years of infant’s life may explain a disparate picture of allergic disease prevalence in Swedish and Estonian populations (45).

### 4.2 The impact of environmental exposures on methylation dynamics in the *IL4*, *IL5*, *IL10*, *IFNG* and *FOXP3* genes

#### 4.2.1 Methylation status and demographic/social data

The potential relationships between the methylation profile of the TH1/TH2 cytokine genes and *FOXP3* and some social data were analysed. The two factors of having no siblings nor animals were correlated with methylation dynamics in all studied groups. For example, Čelakovská et al. (46) reported that persistent AD lesions occurred more often in patients that had become sensitized to animal dander. Allergy to cats, dogs, or both is considered a major risk factor for the development of asthma and rhinitis, and to a lesser extent is associated with atopic dermatitis or some forms of food allergy, however, such associations are controversial [reviewed in (47)]. One could expect that place of residence (village, suburbs or city) may influence the methylation status but we have not found evidence for that. It can be assumed that heavy metals, dust and other pollutants in the air such as particulate matter (PM) are more common in cities. Many studies supported the hypothesis that these factors could influence DNA methylation patterns as reviewed in (48–50). It was shown, for example, that PM2.5 exposure level was positively correlated with methylation level in the *IFNG* promoter region and decreased cytokine expression (51). The cited studies concerned older participants. Perhaps the infants we examined were exposed to these factors for too short, or even protected against them.

The impact of age on methylation processes was also confirmed in this study. We observed that the older the child, the lower was the *IL10* methylation rate in the control group. The same relation between parents’ age and *FOXP3* methylation in controls was detected. Similar but weaker association of mother’s age with methylation of *FOXP3* in allergic infants was noted. On the other hand, in patients with AD, the methylation of *IL4* promoter was increasing along with father’s or mother’s age. A report based on the results of genome-wide DNA methylation profiling of 168 newborns indicated that maternal, and to a lesser extent paternal age contributes to differences in CpG methylation levels at birth. There is a general trend towards hypomethylation of CpG islands, especially in genes associated with oncogenesis and cancer progression in newborn blood cells with increasing parental age (52). It was reported that DNAm gestational age acceleration of the offspring at birth was associated with maternal age of over 40 years at delivery. In contrary, DNAm GA deceleration of the offspring at birth was associated with insulin-treated gestational diabetes mellitus (GDM) in a previous pregnancy and Sjögren’s syndrome. GAA means an older DNAm GA than chronological GA, whereas GAD means younger DNAm GA than chronological GA (53). Older maternal age was significantly associated with reduced methylation at four adjacent CpGs near the second exon of KLHL35 in Norwegian newborns, while no corresponding effect of paternal age on methylation levels at these sites was found (54). Studies on protein expression level of TH1/TH2 genes in children aged 1-96 months demonstrated that the expression of IFNG and IL4 was increasing progressively with age, whereas the levels of IL5 and IL10 tended to be regulated on an individual basis during infancy and early childhood. The IL4 mRNA expression levels were diminished in neonates, then they tended to be increased after birth and remained relatively stable in infancy and childhood. Interestingly, no correlation was observed between IL4 protein expression and IL4 mRNA levels (55). A significant age correlation of IFNG, IL4 and IL5 release in blood samples of atopic and non-atopic children after stimulation with staphylococcal enterotoxin B was reported (56). There are no systemic studies on TH1/TH2 genes methylation alterations with age, but the correlation between methylation of the promoters and mRNA/protein expression levels may be indirect evidence of such a link. A significant influence of age on DNA methylation and mRNA levels of different genes (i.e. ELOV2, HOXC4 and PI4KB) in white blood cells of adults was found (57).

The changes in DNA methylation pattern occur naturally with aging, including global hypomethylation and region-specific hypermethylation. The dynamics of methylation processes is more rapid in children than adults (58), with the majority of changes in childhood taking place during the first five years of life (59). It is well established that DNA methylation biomarkers can determine biological age of any tissue during the development as well as across the entire human lifespan. There is also growing evidence suggesting epigenetic age acceleration to be strongly linked to common diseases or occurring in response to various environmental factors (44, 60). The decrease in DNA methylation level of *IL10* gene associated with infant’s age that we found in control group may be a result of natural maturation process. The lack of such relationship among allergic infants may come from alterations in epigenetic mechanism linked to allergy. The association of changes in *FOXP3* and *IL4* methylation level with parental age is more difficult to elucidate. Epidemiological studies (53, 61, 62) have shown that exposure to adverse environmental events in the prenatal period predicts increased risk of aging-related diseases, which are consistent with the Developmental Origins of Health and Diseases (DOHaD) hypothesis, suggesting that prenatal exposures alter developmental trajectories (63). One option is that placenta epigenetic aging of older mothers is a player as its associations with pre-pregnancy conditions and birth outcomes in children were reported. Accordingly, neonate methylomes contain molecular memory of the individual *in utero* experience (62, 64). It is assumed that each generation begins with a “renewed” epigenome. After fertilization the maternal and paternal genomes undergo a rapid demethylation of most of the genome followed by remethylation. Embryogenesis up until the blastocyst stage is crucial in determining the epigenetic marks needed for later development. The *in utero* period is developmentally plastic life stage, during which environmental exposures may impart long-lasting effects on future phenotype via epigenetic programming. However, there is evidence that the methylation patterns present in the parents are not totally erased and reset during gametogenesis and fertilization at a proportion of loci, including both imprinted and non-imprinted genes (44, 52, 65). So age-related DNA methylation changes also take place in germ cells and might be possibly transmitted to the offspring and may contribute to the increased disease risk in offspring of older parents (52, 66, 67). As information on maternal or paternal DNA methylation status is unavailable, the comparison between parental and offspring’s methylation profile is impossible. The epigenetic inheritance or *in utero* environment effects seem to be important players in shaping the offspring epigenome but other confounding factors cannot be excluded and further studies are needed to clarify this issue.

#### 4.2.2 Cigarette smoking

It was found that methylation levels of some loci declined among infants exposed to second-hand smoke both during pregnancy and after birth. Methylation of *IFNG* was lower within an overall allergic group, methylation of *IL4* and *IL10* was diminished among patients with AD and FA+ADFA. Whereas *FOXP3* exhibited contradictory results depending on the time of smoking exposure. Multiple independent studies on different populations have shown a significant impact of smoking on blood methylome (68). Due to very few cases of active maternal smoke exposures in our study, we resigned from analysing potential links with methylation patterns. However, it is documented that maternal smoking during pregnancy causes many undesirable effects on the offspring including low birth weight, some types of cancers, respiratory dysfunctions, and it leads to obesity and elevated blood pressure (69, 70). Epigenetic mechanisms are suggested since a set of genes with methylation changes present at birth in children whose mothers smoked during pregnancy was identified (71). The association of maternal smoking, birth weight and gestational age with significant DNA methylation differences in neonatal blood was confirmed (72, 73). Functional network analysis suggested a role in activating the immune system (73).

#### 4.2.3 The presence of complications during pregnancy

Interestingly, a link was found between blood methylation status in infants and the occurrence of complications during pregnancy. Abnormal state has been associated with the decrease in methylation of *IFNG*, *IL4*, *IL5* promoters in the healthy group. Also, statistically significant relations for *IL5* in infants with allergy and those with FA or ADFA, as well as for *IL4* in patients with ADFA or FA+ADFA were observed. The presence of pregnancy anomalies was associated with partial demethylation of the analysed loci in infant blood, although not always in a significant manner. These findings are in agreement with other observations of the relationships between maternal environmental exposures during pregnancy and the risk of illness in offspring. The prenatal period is believed to programme epigenetics of foetus, which in turn influences the risk for a range of disorders that develop later in life, including food allergy (examples are discussed (74, 75).

One of the most frequently diagnosed complications of pregnancy in our study was gestational diabetes but in most cases they were accompanied by other disorders like thyroidism, hypertension, bacterial infections and mycoses, and others. Little is known about the biological processes that link the occurrence of these pregnancy complications with adverse child outcomes; altered biological aging of the growing fetus up to birth is one molecular pathway of increasing interest (62). It was shown that maternal dysglycaemia was associated with significant changes in the methylation profile of the infants. Moreover, the epigenetic changes caused by a dysglycaemic prenatal environment appeared to be modifiable by a lifestyle intervention in pregnancy (76). Interestingly, compared to those who did not develop gestational diabetes mellitus, women diagnosed with GDM were older, had a higher BMI, and were more likely to be multiparous (76). As mentioned earlier in this work, DNAm GA deceleration of the offspring at birth was associated with insulin-treated gestational diabetes mellitus (GDM) in a previous pregnancy and Sjögren’s syndrome (53). It was observed that prenatal exposure to gestational diabetes and preeclampsia, but not to hypertension, was associated with reduced biological maturity (decelerated gestational biological age) at birth among female neonates (62). On the other hand, there was evidence that offspring of mothers with hypertensive disorders of pregnancy (compared with those whose mothers who did not experience HDP) had a slightly faster increase in methylation between birth and age 7 (77).

Ours and other researchers’ findings suggest that the epigenetic dynamics is an important biological pathway associated with pregnancy conditions playing a role in mediating the effects of these conditions on perinatal and child health outcomes. Further research on the functional relevance of methylation changes at specific sites is required.

#### 4.2.4 The type of infant feeding

Some differences in methylation profiles were shown to be associated with the type of feeding within groups of allergic and healthy infants. In the case of exclusive breastfeeding, demethylation of *IFNG* was observed among patients with FA, whereas hypermethylation was characteristic for infants with AD. Contradictory results were obtained for infants exclusively fed with milk formula. Additionally, breastfeeding seemed to be linked with a lower *IL5* methylation rate in AD infants. There is no consensus among researchers about the effects of diet during pregnancy and in early childhood on allergy development. It was suggested that observed discrepancies in the results which were varying from a protective or neutral effect to a disease-promoting effect can result from other modulating factors affecting the immunological processes, for example age, gender, family history of allergies, coexistence of other allergies or genetic background (74). We have also observed the influence of some of these factors on the methylation dynamics as is discussed elsewhere in this work. It has been confirmed that improving the diet and exercise influence methylation biomarkers of healthy aging (78). Exclusive breastfeeding is suggested to prevent the risks of overweight/obesity of children and adults through DNA methylation mechanisms occurring early in life (79). Modifications of methylation patterns in blood playing important roles in various biological pathways including development and function of the immune system have also been associated with the duration of breastfeeding (80). L-Arginine is an important nutrient in the infant diet that by methylation mechanisms is acting as a regulator of the maturation process of the immune system in neonates, including the maturation of CD4+ T cells. Interestingly, after L-arginine treatment, more CpG dinucleotides were hypomethylated and more genes appeared to be activated in neonatal T-cells as compared with adult (76). Besides these findings, the role of breastfeeding in modulating the epigenetic pathways remains unclear. Introducing milk formula into the child’s diet and maternal supplementation during breastfeeding are suggested to influence baby’s methylome. Prenatal *L. reuteri* supplementation was shown to change DNA methylation patterns in CD4+ T cells of newborns enhancing immune activation at birth, resulting in affecting immune maturation and allergy development (81). It was observed that hydrolysed casein formula containing probiotics for infants drew higher *IL4*/*IL5* and lower *IL10*/*IFNG* DNA methylation patterns in CD4+ T cells than soya formula (30). L-arginine treatment induced greater hypomethylation of CpG dinucleotides and gene activation in neonatal T-cells as compared with adult. Among the most stimulated were genes regarding immune-related pathways (30). The L-arginine intake influenced the *IL10* promoter DNA hypomethylation inducing increase in IL10 production by neonatal CD4+ T cells (82). ω-3 PUFA seemed to have immunomodulatory properties via modifying DNA methylation since altered LINE1 repetitive sequences’ methylation in children of women who smoked during pregnancy, as well as modulated DNA methylation levels in the promoters of genes encoding IFNG and IL13 were observed (83). Vitamin C was found to prevent offspring DNA methylation changes associated with maternal smoking during pregnancy (84). Furthermore, gestational vitamin D deficiency is considered to increase IL4 concentration and decrease the Th1/Th2 ratio and IFNG production. The maternal deficit of vitamin D was shown to cause an increase in the activity of DNA methyltransferase and hypermethylation of *IFNG* locus. The process can be reversed by vitamin D supplementation during pregnancy (85). Acevedo et al. (86) compiled studies on the effects of breastfeeding on the onset and course of allergic diseases. The authors indicated that some findings have supported the protective effects of breastfeeding on food allergy, atopic dermatitis and overall positive effect on gut and respiratory health, while some of them showed no or even an increased risk in children who were breastfed. The inconsistencies between studies are most likely due to differences in duration of breastfeeding, the amount of milk given, genetic predisposition and methodology. One cannot ignore the fact that the composition of milk may differ between individuals and across populations, and it may depend on maternal nutritional status, smoking, having pets, and geographic location, etc.

#### 4.2.5 BMI/weight of mother or infant

We also demonstrated the effects of birth/current weight of the child as well as mother BMI on CpG methylation status that is consistent with other findings. It was reported elsewhere that pre-gestational maternal obesity contributed to decreased basal expression of pro-inflammatory mediators in monocytes and in the latter, to suppressed expression of the key inflammatory regulator IL10 in activated macrophages. Alterations in the expression of *IL-1β* and *IL10* were associated with DNA methylation dynamics in their promoter regions (87). We found that with an increase in maternal BMI/weight value DNA methylation of some loci in offspring blood has become lower. Changes toward demethylation of *IFNG* and *IL5* associated with higher BMI were observed in controls and of *IL10* in allergic infants. A similar negative correlation appeared for mother’s weight and *IL5* methylation status in healthy infants. There are many reports confirming positive or negative correlations, depending on the locus, between maternal BMI/adiposity and DNA methylation in neonates (88–95). Lifestyle interventions in pregnant women with obesity contributed to epigenetic changes in offspring, potentially influencing its lean mass and early growth (96).

Additionally, we have found that the *IL10* methylation level was increasing along with a child’s birth weight in a group consisting of all patients as well as FA+ADFA participants. Similarly, the current child’s weight was positively correlated with the *IL10* methylation level in allergic infants. DNA hypermethylation found in cells of blood or adipose tissue of obese individuals is the consequence of obesity (97). In another study, the association between increased BMI and accelerated epigenetic aging in the blood cells of middle-aged individuals was noted (98).

The results of epigenome-wide association studies (EWAS) regarding paternal prenatal body mass index (BMI) in relation to DNA methylation in offspring blood at birth and in childhood do not confirm any association between these variables, even at imprinted regions (88, 99). It supports the hypothesis of the uterine microenvironment playing a role in methylation shaping.

#### 4.2.6 Family history of allergy

We detected a weak positive correlation between the number of allergic family members and *IL4* promoter methylation within certain subgroups such as the healthy group, patients with AD and ADFA or with AD only. It seems likely that history of familial allergy may increase the risk of allergy symptoms in the next child. The heritability of epigenetic marks and their association with DNA variants were documented (100–102). A neonatal epigenome is shaped by both intrauterine environmental and underlying genetic factors (100). It is the evidence that allele-specific DNA methylation is widespread across the genome, most of which can be strongly influenced by the sequence of adjacent SNPs (101, 102). Average heritability for DNA methylation is estimated at approximately 20% (103). There are studies indicating food allergies heritability estimates ranging from 15% to 30% for food specific IgE (104). The recent rise in prevalence of food allergies suggests a low to moderate impact of genetic factors and highlights the environmental exposure issues. Only a proportion of children subjected to variation in these exposures develop clinical food allergy, suggesting that some individuals are more genetically susceptible than others, but only in certain environments (23). The sequence information is inherited from the parents but epigenetic pattern is arising *de novo* during development of the embryo, except for a group of promoter sequences (CpG islands) needed for the expression of the developmental genes (105). On the other hand, research on animal models indicated that epigenetic patterns affected by external factors can persist across generations depending upon the timing of exposures (23). The genes are capable of ‘learning’ or ‘adapting’ to the environment over time and highlighted that the very complex interplay between genetic and ambient components during early immune development is at the root of the allergy (23). The risk of FA among one-year-old allergic infants was higher when there was any allergy in one immediate member of family and increased even to 80% when two such members were present in comparison to children without family history of allergy (106).

Limitations of this study include a low number of participants, especially patients with AD and the fact that we tested the blood of infants with active allergic symptoms at the time of inclusion, in some of them the initial diagnosis was verified. The age may be a weakness of this study in terms of establishing a diagnosis, but it was also a strong point. The knowledge on DNA methylation mechanisms in the food allergy/atopic dermatitis development in infants is obscure. Our research partially fills the gap because we test the blood of infants at a time when the infants methylome begins to form outside the womb in response to external factors. Besides, the comparisons between infants of similar age is very beneficial. Additionally, the effects of cell mixture on the measurement of DNA methylation in whole blood must be considered. On the other hand, counting of cell type may also be predictive in allergic processes. More extensive validation of methylation analysis of whole blood is required.

In conclusion, the potential of examination of a few drops of whole blood to detect methylation changes in the immune-attached genes using a cost-effective MS-HRM PCR methodology was demonstrated. It was found that methylation rate in *FOXP3* TSDR region in allergic infants was lower than in the healthy ones. It was also shown that methylation profile of *IL4*, *IL5*, *IL10*, *IFNG* and *FOXP3* was associated with environmental exposures and the direction of methylation dynamics differed depending on the presence and types of allergy symptoms. These results indicate that the interpretation of methylation pattern in the context of an allergic disease should take into account ambient factors having impact on epigenetic variation in early childhood. Undoubtedly, the development and maturation of the immune system is a very complicated process influenced by both individual genetic background and the environmental exposures regulating action of different types of cells. Further studies are needed to clarify this network.

## Data availability statement

The raw data supporting the conclusions of this article will be made available by the authors, without undue reservation.

## Ethics statement

The studies involving human participants were reviewed and approved by Bioethics Committee of Ludwik Rydygier Collegium Medicum in Bydgoszcz Nicolaus Copernicus University in Toruń, Poland. Written informed consent to participate in this study was provided by the participants’ legal guardian/next of kin.

## Author contributions

AK, EŁ-R, MG contributed to the conception, design, data collection, data analysis, and manuscript writing. MG was responsible for statistical analyses and interpretation and drafted the manuscript. JG contributed to the data collection. TG supervised the study. TG, MGo helped with manuscript writing and editing. All authors contributed to the article and approved the submitted version.
