# Supplementary material for: The methylation profile of IL4, IL5, IL10, IFNG and FOXP3 associated with environmental exposures differed between Polish infants with the food allergy and/or atopic dermatitis and without the disease
Source: Front Immunol. 2023 Jul 13;14:1209190. doi: 10.3389/fimmu.2023.1209190 (PMC10373304; doi:10.3389/fimmu.2023.1209190)
Supplement: Supplementary file 1 [file Table_1.docx]

| Locus | Primers sequence (5’-3’) |
| --- | --- |
| IL4* | Forward methylated: GTGTCGATTTGTAGTGATAATGTGA  Reverse methylated: AACGAAAATTTCCAATATAAACTCAT |
| IL5 | Forward methylated: TCGTCGTATCGTATTCGGAAGTC  Reverse methylated: GAATCACGTAAACCTCTAAACCCG  Forward unmethylated: TTGTTGTATTGTATTTGGAAGTTGG  Reverse unmethylated: AAATCACATAAACCTCTAAACCCACA |
| IL10 | Forward methylated: TGCGTAGAGGTTTTTAGTTGTGG  Reverse methylated: TTCTAAAATAAACAATTTATCCACGTCA  Forward unmethylated: TTGTGTGTAGAGGTTTTTAGTTGTGG  Reverse unmethylated: TAAAATAAACAATTTATCCACATCACT |
| IFNG** | Forward: TGGGTTTTGGTAGTAATAGTTAAGAG  Reverse: ACAAAAACTACTAATTTCAACTTCTTTA |
| FOXP3*** | Forward methylated: GTTTTCGATTTGTTTAGATTTTTTCGTT  Reverse methylated: CCTCTTCTCTTCCTCCGTAATATCG  Forward methylated: GTTTTTGATTTGTTTAGATTTTTTTGTT  Reverse unmethylated: CCTCTTCTCTTCCTCCATAATATCA |

Table S1. The primers used for analysis of CpG sites.

* - primers design was successful for only methylated sequences

** - primers for methylated and unmethylated sequences were identical

*** - primers from (26)
