# Supplementary material for: The methylation profile of IL4, IL5, IL10, IFNG and FOXP3 associated with environmental exposures differed between Polish infants with the food allergy and/or atopic dermatitis and without the disease
Source: Front Immunol. 2023 Jul 13;14:1209190. doi: 10.3389/fimmu.2023.1209190 (PMC10373304; doi:10.3389/fimmu.2023.1209190)
Supplement: Supplementary file 2 [file Table_2.docx]

| Locus | Variable | Control group | | Allergic group | | FA | | AD | | ADFA | | FA+ADFA | | AD+ADFA | |
| --- | --- | --- | --- | --- | --- | --- | --- | --- | --- | --- | --- | --- | --- | --- | --- |
|  |  | H_K-W_ | p | H_K-W_ | p | H_K-W_ | p | H_K-W_ | p | H_K-W_ | p | H_K-W_ | p | H_K-W_ | p |
| IL4 | Gender | 0.069 | 0.793 | 0.028 | 0.868 | 1.066 | 0.302 | 0.058 | 0.810 | 1.056 | 0.304 | 0.066 | 0.797 | 0.784 | 0.376 |
| IL5 |  | 0.257 | 0.612 | 0.052 | 0.819 | 1.320 | 0.251 | 0.004 | 0.949 | 0.287 | 0.592 | 0.037 | 0.848 | 0.203 | 0.652 |
| IL10 |  | 0.019 | 0.889 | 1.037 | 0.309 | 0.560 | 0.454 | 0.014 | 0.906 | 0.502 | 0.479 | 1.241 | 0.265 | 0.366 | 0.545 |
| IFNG |  | 0.445 | 0.505 | 0.743 | 0.389 | 1.615 | 0.204 | 0.037 | 0.848 | 3.827 | 0.050 | 0.740 | 0.390 | 3.408 | 0.062 |
| FOXP3 |  | 0.062 | 0.803 | 0.339 | 0.560 | 1.844 | 0.174 | 0.237 | 0.626 | 0.077 | 0.782 | 0.661 | 0.416 | 0.000 | 0.997 |

Table S2. The association between DNA methylation level of the *IL4*, *IL5*, *IL10*, *IFNG* and *FOXP3* loci and infant’s gender. C – control group, A – allergic group, FA – group with food allergy, AD – group with atopic dermatitis, ADFA – group with atopic dermatitis and food allergy, H_K-W_ – Kruskal-Wallis ANOVA coefficient, level of significance p<0.05.
