# Supplementary material for: The methylation profile of IL4, IL5, IL10, IFNG and FOXP3 associated with environmental exposures differed between Polish infants with the food allergy and/or atopic dermatitis and without the disease
Source: Front Immunol. 2023 Jul 13;14:1209190. doi: 10.3389/fimmu.2023.1209190 (PMC10373304; doi:10.3389/fimmu.2023.1209190)
Supplement: Supplementary file 3 [file Table_3.docx]

| Locus | Variable | Control group | | Allergic group | | FA | | AD | | ADFA | | FA+ADFA | | AD+ADFA | |
| --- | --- | --- | --- | --- | --- | --- | --- | --- | --- | --- | --- | --- | --- | --- | --- |
|  |  | H_K-W_ | p | H_K-W_ | p | H_K-W_ | p | H_K-W_ | p | H_K-W_ | p | H_K-W_ | p | H_K-W_ | p |
| IL4 | Having siblings | 0.005 | 0.944 | 0.531 | 0.466 | 0.008 | 0.929 | 2.074 | 0.150 | 0.155 | 0.693 | 0.078 | 0.780 | 0.849 | 0.357 |
| IL5 |  | 1.180 | 0.277 | 0.430 | 0.512 | 0.473 | 0.491 | 1.977 | 0.160 | 0.602 | 0.438 | 0.032 | 0.858 | 1.609 | 0.205 |
| IL10 |  | 0.107 | 0.743 | 0.273 | 0.601 | 2.099 | 0.147 | 0.500 | 0.480 | 2.037 | 0.154 | 0.121 | 0.728 | 2.357 | 0.125 |
| IFNG |  | 1.062 | 0.303 | 0.268 | 0.605 | 2.022 | 0.155 | 0.037 | 0.848 | 0.206 | 0650 | 0.262 | 0.608 | 0.128 | 0.721 |
| FOXP3 |  | 0.072 | 0.788 | 0.354 | 0.552 | 0.001 | 0.975 | 0.033 | 0.855 | 0.393 | 0.531 | 0.276 | 0.599 | 0.504 | 0.478 |

Table S3. The association between DNA methylation level of the *IL4*, *IL5*, *IL10*, *IFNG* and *FOXP3* loci and having siblings. C – control group, A – allergic group, FA – group with food allergy, AD – group with atopic dermatitis, ADFA – group with atopic dermatitis and food allergy, H_K-W_ – Kruskal-Wallis ANOVA coefficient, level of significance p<0.05.
