# Supplementary material for: The methylation profile of IL4, IL5, IL10, IFNG and FOXP3 associated with environmental exposures differed between Polish infants with the food allergy and/or atopic dermatitis and without the disease
Source: Front Immunol. 2023 Jul 13;14:1209190. doi: 10.3389/fimmu.2023.1209190 (PMC10373304; doi:10.3389/fimmu.2023.1209190)
Supplement: Supplementary file 4 [file Table_4.docx]

Table S4. The association between DNA methylation level of the *IL4*, *IL5*, *IL10*, *IFNG* and *FOXP3* loci and number of siblings. C – control group, A – allergic group, FA – group with food allergy, AD – group with atopic dermatitis, ADFA – group with atopic dermatitis and food allergy, rho – Spearmans’ rho coefficient, level of significance p<0.05.

| Locus | Variable | Control group | | Allergic group | | FA | | AD | | ADFA | | FA+ADFA | | AD+ADFA | |
| --- | --- | --- | --- | --- | --- | --- | --- | --- | --- | --- | --- | --- | --- | --- | --- |
|  |  | rho | p | rho | p | rho | p | rho | p | rho | p | rho | p | rho | p |
| IL4 | Number of siblings | -0.001 | 0.994 | 0.071 | 0.410 | 0.045 | 0.788 | 0.378 | 0.165 | 0.035 | 0.752 | 0.038 | 0.675 | 0.082 | 0.418 |
| IL5 |  | -0.116 | 0.281 | 0.030 | 0.725 | -0.162 | 0.332 | 0.358 | 0.190 | 0.070 | 0.524 | -0.010 | 0.913 | 0.113 | 0.263 |
| IL10 |  | -0.035 | 0.748 | -0.016 | 0.593 | 0.242 | 0.143 | -0.136 | 0.629 | -0.178 | 0.104 | -0.040 | 0.659 | -0.164 | 0.104 |
| IFNG |  | 0.103 | 0.337 | 0.043 | 0.613 | 0.284 | 0.084 | 0.020 | 0.943 | -0.069 | 0.528 | 0.050 | 0.580 | -0.059 | 0.559 |
| FOXP3 |  | -0.035 | 0.747 | 0.026 | 0.763 | -0.018 | 0.914 | 0.032 | 0.910 | 0.038 | 0.732 | 0.024 | 0.793 | 0.040 | 0.692 |
