# Supplementary material for: The methylation profile of IL4, IL5, IL10, IFNG and FOXP3 associated with environmental exposures differed between Polish infants with the food allergy and/or atopic dermatitis and without the disease
Source: Front Immunol. 2023 Jul 13;14:1209190. doi: 10.3389/fimmu.2023.1209190 (PMC10373304; doi:10.3389/fimmu.2023.1209190)
Supplement: Supplementary file 5 [file Table_5.docx]

| Locus | Variable | Control group | | Allergic group | | FA | | AD | | ADFA | | FA+ADFA | | AD+ADFA | |
| --- | --- | --- | --- | --- | --- | --- | --- | --- | --- | --- | --- | --- | --- | --- | --- |
|  |  | H_K-W_ | p | H_K-W_ | p | H_K-W_ | p | H_K-W_ | p | H_K-W_ | p | H_K-W_ | p | H_K-W_ | p |
| IL4 | Having animals | 1.738 | 0.187 | 0.175 | 0.675 | 0.986 | 0.321 | 1.389 | 0.239 | 0.225 | 0.635 | 0.002 | 0.963 | 0.025 | 0.875 |
| IL5 |  | 2.126 | 0.145 | 0.096 | 0.757 | 0.138 | 0.711 | 0.477 | 0.490 | 0.622 | 0.430 | 0.394 | 0.530 | 0.181 | 0.670 |
| IL10 |  | 0.003 | 0.959 | 0.200 | 0.655 | 0.769 | 0.380 | 0.013 | 0.908 | 0.000 | 1.000 | 0.136 | 0.712 | 0.013 | 0.910 |
| IFNG |  | 0.686 | 0.407 | 0.228 | 0.633 | 0.766 | 0.382 | 0.016 | 0.900 | 1.642 | 0.200 | 0.258 | 0.611 | 1.281 | 0.258 |
| FOXP3 |  | 0.009 | 0.925 | 0.001 | 0.971 | 0.804 | 0.370 | 0.289 | 0.591 | 0.175 | 0.676 | 0.016 | 0.900 | 0.198 | 0.656 |

Table S5. The association between DNA methylation level of the *IL4*, *IL5*, *IL10*, *IFNG* and *FOXP3* loci and having animals. C – control group, A – allergic group, FA – group with food allergy, AD – group with atopic dermatitis, ADFA – group with atopic dermatitis and food allergy, H_K-W_ – Kruskal-Wallis ANOVA coefficient, level of significance p<0.05.
