# Supplementary material for: The methylation profile of IL4, IL5, IL10, IFNG and FOXP3 associated with environmental exposures differed between Polish infants with the food allergy and/or atopic dermatitis and without the disease
Source: Front Immunol. 2023 Jul 13;14:1209190. doi: 10.3389/fimmu.2023.1209190 (PMC10373304; doi:10.3389/fimmu.2023.1209190)
Supplement: Supplementary file 6 [file Table_6.docx]

Table S6. The association between DNA methylation level of the *IL4*, *IL5*, *IL10*, *IFNG* and *FOXP3* loci and number of animals. C – control group, A – allergic group, FA – group with food allergy, AD – group with atopic dermatitis, ADFA – group with atopic dermatitis and food allergy, rho – Spearmans’ rho coefficient, level of significance p<0.05.

| Locus | Variable | Control group | | Allergic group | | FA | | AD | | ADFA | | FA+ADFA | | AD+ADFA | |
| --- | --- | --- | --- | --- | --- | --- | --- | --- | --- | --- | --- | --- | --- | --- | --- |
|  |  | rho | p | rho | p | rho | p | rho | p | rho | p | rho | p | rho | p |
| IL4 | Number of animals | 0.143 | 0.182 | -0.037 | 0.665 | -0.184 | 0.269 | -0.382 | 0.160 | 0.050 | 0.647 | -0.002 | 0.985 | -0.015 | 0.886 |
| IL5 |  | 0.149 | 0.164 | 0.030 | 0.724 | 0.008 | 0.964 | -0.227 | 0.415 | 0.072 | 0.513 | 0.062 | 0.496 | 0.027 | 0.790 |
| IL10 |  | -0.005 | 0.960 | -0.070 | 0.413 | -0.136 | 0.414 | -0.092 | 0.745 | -0.034 | 0.758 | -0.063 | 0.490 | -0.047 | 0.641 |
| IFNG |  | 0.099 | 0.357 | 0.034 | 0.695 | -0.112 | 0.502 | -0.039 | 0.890 | 0.132 | 0.229 | 0.045 | 0.624 | 0.101 | 0.319 |
| FOXP3 |  | -0.015 | 0.890 | -0.008 | 0.922 | -0.108 | 0.519 | 0.097 | 0.731 | 0.036 | 0.746 | -0.012 | 0.899 | 0.031 | 0.761 |
