# Supplementary material for: The methylation profile of IL4, IL5, IL10, IFNG and FOXP3 associated with environmental exposures differed between Polish infants with the food allergy and/or atopic dermatitis and without the disease
Source: Front Immunol. 2023 Jul 13;14:1209190. doi: 10.3389/fimmu.2023.1209190 (PMC10373304; doi:10.3389/fimmu.2023.1209190)
Supplement: Supplementary file 7 [file Table_7.docx]

| Locus | Variable | Control group | | Allergic group | | FA | | AD | | ADFA | | FA+ADFA | | AD+ADFA | |
| --- | --- | --- | --- | --- | --- | --- | --- | --- | --- | --- | --- | --- | --- | --- | --- |
|  |  | H_K-W_ | p | H_K-W_ | p | H_K-W_ | p | H_K-W_ | p | H_K-W_ | p | H_K-W_ | p | H_K-W_ | p |
| IL4 | Place of residence | 0.539 | 0.764 | 0.789 | 0.674 | 0.907 | 0.636 | 1.159 | 0.560 | 1.271 | 0.530 | 2.019 | 0.364 | 0.240 | 0.887 |
| IL5 |  | 0.064 | 0.969 | 1.850 | 0.397 | 0.234 | 0.890 | 1.930 | 0.381 | 2.356 | 0.308 | 1.059 | 0.589 | 2.746 | 0.253 |
| IL10 |  | 3.131 | 0.209 | 0.152 | 0.927 | 0.101 | 0.951 | 1.120 | 0.571 | 0.494 | 0.781 | 0.228 | 0.892 | 0.270 | 0.874 |
| IFNG |  | 0.894 | 0.640 | 1.749 | 0.417 | 2.151 | 0.341 | 0.582 | 0.747 | 1.376 | 0.503 | 3.292 | 0.193 | 0.376 | 0.829 |
| FOXP3 |  | 2.064 | 0.356 | 0.851 | 0.653 | 0.964 | 0.618 | 2.602 | 0.272 | 0.375 | 0.829 | 1.205 | 0.547 | 0.642 | 0.725 |

Table S7. The association between DNA methylation level of the *IL4*, *IL5*, *IL10*, *IFNG* and *FOXP3* loci and place of residence. Inhabitants of city, suburbs and village were taken into account. C – control group, A – allergic group, FA – group with food allergy, AD – group with atopic dermatitis, ADFA – group with atopic dermatitis and food allergy, H_K-W_ – Kruskal-Wallis ANOVA coefficient, level of significance p<0.05.
