# Supplementary material for: The methylation profile of IL4, IL5, IL10, IFNG and FOXP3 associated with environmental exposures differed between Polish infants with the food allergy and/or atopic dermatitis and without the disease
Source: Front Immunol. 2023 Jul 13;14:1209190. doi: 10.3389/fimmu.2023.1209190 (PMC10373304; doi:10.3389/fimmu.2023.1209190)
Supplement: Supplementary file 8 [file Table_8.docx]

Table S8. The association between DNA methylation level of the *IL4*, *IL5*, *IL10*, *IFNG* and *FOXP3* loci and age of mother, father or infant. C – control group, A – allergic group, FA – group with food allergy, AD – group with atopic dermatitis, ADFA – group with atopic dermatitis and food allergy, rho – Spearmans’ rho coefficient, level of significance p<0.05.

| Locus | Variable | Control group | | Allergic group | | FA | | AD | | ADFA | | FA+ADFA | | AD+ADFA | |
| --- | --- | --- | --- | --- | --- | --- | --- | --- | --- | --- | --- | --- | --- | --- | --- |
|  |  | rho | p | rho | p | rho | p | rho | p | rho | p | rho | p | rho | p |
| IL4 | Mother’s age | -0.187 | 0.079 | 0.035 | 0.680 | -0.054 | 0.749 | 0.569 | 0.027 | -0.010 | 0.930 | -0.030 | 0.745 | 0.077 | 0.449 |
| IL5 |  | -0.122 | 0.253 | 0.000 | 1.000 | -0.285 | 0.083 | 0.256 | 0.356 | 0.120 | 0.273 | -0.034 | 0.708 | 0.130 | 0.198 |
| IL10 |  | -0.204 | 0.055 | -0.053 | 0.540 | 0.005 | 0.976 | -0.077 | 0.784 | -0.091 | 0.406 | -0.060 | 0.508 | -0.075 | 0.458 |
| IFNG |  | -0.144 | 0.178 | 0.057 | 0.507 | 0.156 | 0.349 | 0.096 | 0.735 | 0.002 | 0.989 | 0.050 | 0.583 | 0.020 | 0.847 |
| FOXP3 |  | -0.229 | 0.031 | -0.056 | 0.514 | -0.019 | 0.910 | 0.242 | 0.384 | -0.136 | 0.216 | -0.101 | 0.267 | -0.063 | 0.536 |
| IL4 | Father’s age | -0.022 | 0.838 | 0.084 | 0.326 | 0.064 | 0.704 | 0.521 | 0.047 | 0.032 | 0.775 | 0.028 | 0.762 | 0.109 | 0.279 |
| IL5 |  | -0.123 | 0.249 | 0.029 | 0.731 | -0.105 | 0.532 | 0.290 | 0.295 | 0.076 | 0.491 | -0.008 | 0.926 | 0.108 | 0.285 |
| IL10 |  | -0.096 | 0.369 | -0.013 | 0.884 | 0.052 | 0.757 | 0.016 | 0.954 | -0.012 | 0.916 | -0.008 | 0.933 | -0.020 | 0.841 |
| IFNG |  | -0.131 | 0.221 | 0.035 | 0.685 | 0.068 | 0.686 | 0.012 | 0.967 | 0.009 | 0.932 | 0.038 | 0.673 | 0.012 | 0.908 |
| FOXP3 |  | -0.268 | 0.011 | -0.018 | 0.832 | -0.183 | 0.272 | 0.303 | 0.272 | -0.037 | 0.734 | -0.072 | 0.430 | 0.036 | 0.723 |
| IL4 | Child’s age | 0.160 | 0.135 | 0.029 | 0.739 | -0.042 | 0.804 | 0.029 | 0.918 | 0.066 | 0.547 | 0.036 | 0.690 | 0.054 | 0.592 |
| IL5 |  | -0.051 | 0.633 | 0.057 | 0.510 | 0.111 | 0.506 | -0.197 | 0.481 | 0.096 | 0.380 | 0.091 | 0.317 | 0.051 | 0.611 |
| IL10 |  | -0.300 | 0.004 | 0.096 | 0.262 | 0.109 | 0.513 | -0.142 | 0.614 | 0.114 | 0.301 | 0.121 | 0.182 | 0.084 | 0.408 |
| IFNG |  | 0.014 | 0.898 | 0.033 | 0.705 | 0.000 | 0.081 | 0.161 | 0.565 | 0.127 | 0.248 | 0.010 | 0.910 | 0.137 | 0.174 |
| FOXP3 |  | 0.080 | 0.456 | -0.050 | 0.564 | -0.171 | 0.304 | -0.088 | 0.755 | 0.021 | 0.850 | -0.035 | 0.697 | -0.014 | 0.887 |
