# Supplementary material for: The methylation profile of IL4, IL5, IL10, IFNG and FOXP3 associated with environmental exposures differed between Polish infants with the food allergy and/or atopic dermatitis and without the disease
Source: Front Immunol. 2023 Jul 13;14:1209190. doi: 10.3389/fimmu.2023.1209190 (PMC10373304; doi:10.3389/fimmu.2023.1209190)
Supplement: Supplementary file 9 [file Table_9.docx]

| Locus | Variable | Control group | | Allergic group | | FA | | AD | | ADFA | | FA+ADFA | | AD+ADFA | |
| --- | --- | --- | --- | --- | --- | --- | --- | --- | --- | --- | --- | --- | --- | --- | --- |
|  |  | H_K-W_ | p | H_K-W_ | p | H_K-W_ | p | H_K-W_ | p | H_K-W_ | p | H_K-W_ | p | H_K-W_ | p |
| IL4 | Maternal passive smoke exposure during pregnancy | 0.530 | 0.467 | 1.889 | 0.169 | 0.210 | 0.647 | 6.381 | 0.012 | 0.226 | 0.635 | 0.229 | 0.632 | 2.399 | 0.121 |
| IL5 |  | 0.136 | 0.713 | 0.639 | 0.424 | 0.016 | 0.899 | 0.406 | 0.524 | 1.077 | 0.299 | 0.340 | 0.560 | 1.510 | 0.219 |
| IL10 |  | 0.053 | 0.818 | 1.489 | 0.222 | 1.486 | 0.223 | 0.153 | 0.695 | 0.674 | 0.412 | 2.108 | 0.147 | 0.323 | 0.570 |
| IFNG |  | 1.296 | 0.255 | 5.785 | 0.016 | 2.667 | 0.102 | 1.810 | 0.179 | 1.630 | 0.202 | 4.130 | 0.042 | 3.194 | 0.074 |
| FOXP3 |  | 2.102 | 0.147 | 0.008 | 0.928 | 0.460 | 0.498 | 5.255 | 0.022 | 3.019 | 0.082 | 0.895 | 0.344 | 0.133 | 0.715 |
| IL4 | Infant passive smoke exposure | 0.046 | 0.829 | 1.512 | 0.219 | 0.210 | 0.647 | 3.125 | 0.077 | 0.023 | 0.879 | 0.064 | 0.800 | 1.509 | 0.219 |
| IL5 |  | 0.674 | 0.412 | 0.032 | 0.858 | 0.016 | 0.899 | 0.394 | 0.530 | 0.069 | 0.793 | 0.136 | 0.713 | 0.030 | 0.862 |
| IL10 |  | 0.030 | 0.862 | 2.445 | 0.118 | 1.486 | 0.223 | 1.621 | 0.203 | 0.330 | 0.068 | 5.116 | 0.024 | 1.028 | 0.311 |
| IFNG |  | 0.001 | 0.977 | 5.359 | 0.021 | 2.667 | 0.102 | 1.276 | 0.259 | 1.476 | 0.224 | 3.804 | 0.051 | 2.966 | 0.085 |
| FOXP3 |  | 0.323 | 0.570 | 0.097 | 0.755 | 0.460 | 0.498 | 2.604 | 0.107 | 4.670 | 0.031 | 1.854 | 0.173 | 0.559 | 0.455 |

Table S9. The association between DNA methylation level of the *IL4*, *IL5*, *IL10*, *IFNG* and *FOXP3* loci and passive smoking exposure of infant. C – control group, A – allergic group, FA – group with food allergy, AD – group with atopic dermatitis, ADFA – group with atopic dermatitis and food allergy, H_K-W_ – Kruskal-Wallis ANOVA coefficient, level of significance p<0.05.
