# Supplementary material for: The methylation profile of IL4, IL5, IL10, IFNG and FOXP3 associated with environmental exposures differed between Polish infants with the food allergy and/or atopic dermatitis and without the disease
Source: Front Immunol. 2023 Jul 13;14:1209190. doi: 10.3389/fimmu.2023.1209190 (PMC10373304; doi:10.3389/fimmu.2023.1209190)
Supplement: Supplementary file 10 [file Table_10.docx]

| Locus | Variable | Control group | | Allergic group | | FA | | AD | | ADFA | | FA+ADFA | | AD+ADFA | |
| --- | --- | --- | --- | --- | --- | --- | --- | --- | --- | --- | --- | --- | --- | --- | --- |
|  |  | H_K-W_ | p | H_K-W_ | p | H_K-W_ | p | H_K-W_ | p | H_K-W_ | p | H_K-W_ | p | H_K-W_ | p |
| IL4 | Complications during pregnancy | 1.133 | 0.287 | 3.367 | 0.067 | 0.512 | 0.474 | 0.681 | 0.409 | 4.301 | 0.038 | 4.118 | 0.042 | 3.191 | 0.074 |
| IL5 |  | 5.231 | 0.022 | 5.298 | 0.021 | 3.688 | 0.055 | 0.567 | 0.451 | 2.326 | 0.127 | 5.131 | 0.024 | 2.163 | 0.141 |
| IL10 |  | 4.618 | 0.032 | 2.226 | 0.136 | 2.110 | 0.146 | 0.000 | 1.000 | 1.132 | 0.287 | 2.502 | 0.114 | 0.903 | 0.342 |
| IFNG |  | 5.828 | 0.016 | 0.847 | 0.358 | 1.356 | 0.244 | 0.772 | 0.380 | 0.437 | 0.509 | 1.622 | 0.203 | 0.078 | 0.780 |
| FOXP3 |  | 1.242 | 0.265 | 0.028 | 0.866 | 0.009 | 0.925 | 0.804 | 0.370 | 0.278 | 0.598 | 0.192 | 0.662 | 0.049 | 0.824 |

Table S10. The association between DNA methylation level of the *IL4*, *IL5*, *IL10*, *IFNG* and *FOXP3* loci and complications during pregnancy. C – control group, A – allergic group, FA – group with food allergy, AD – group with atopic dermatitis, ADFA – group with atopic dermatitis and food allergy, H_K-W_ – Kruskal-Wallis ANOVA coefficient, level of significance p<0.05.
