# Supplementary material for: The methylation profile of IL4, IL5, IL10, IFNG and FOXP3 associated with environmental exposures differed between Polish infants with the food allergy and/or atopic dermatitis and without the disease
Source: Front Immunol. 2023 Jul 13;14:1209190. doi: 10.3389/fimmu.2023.1209190 (PMC10373304; doi:10.3389/fimmu.2023.1209190)
Supplement: Supplementary file 11 [file Table_11.docx]

| Locus | Variable | Control group | | Allergic group | | FA | | AD | | ADFA | | FA+ADFA | | AD+ADFA | |
| --- | --- | --- | --- | --- | --- | --- | --- | --- | --- | --- | --- | --- | --- | --- | --- |
|  |  | H_K-W_ | p | H_K-W_ | p | H_K-W_ | p | H_K-W_ | p | H_K-W_ | p | H_K-W_ | p | H_K-W_ | p |
| IL4 | Excusively breastfeeding | 0.521 | 0.470 | 0.168 | 0.682 | 0.425 | 0.514 | 0.014 | 0.904 | 0.006 | 0.937 | 0.197 | 0.657 | 0.004 | 0.951 |
| IL5 |  | 0.210 | 0.647 | 0.108 | 0.743 | 1.760 | 0.185 | 3.926 | 0.048 | 2.459 | 0.117 | 0.140 | 0.708 | 0.329 | 0.567 |
| IL10 |  | 0.291 | 0.590 | 1.677 | 0.195 | 0.018 | 0.893 | 1.389 | 0.239 | 0.889 | 0.346 | 1.108 | 0.293 | 1.545 | 0.214 |
| IFNG |  | 1.308 | 0.253 | 0.280 | 0.597 | 3.139 | 0.076 | 4.449 | 0.035 | 1.020 | 0.312 | 0.058 | 0.810 | 3.373 | 0.066 |
| FOXP3 |  | 0.000 | 1.000 | 0.434 | 0.510 | 0.445 | 0.505 | 0.300 | 0.584 | 0.556 | 0.456 | 0.842 | 0.359 | 0.176 | 0.675 |
| IL4 | Excusively milk formula- feeding | 0.002 | 0.969 | 0.142 | 0.706 | 0.755 | 0.385 | 0.222 | 0.637 | 0.019 | 0.891 | 0.446 | 0.504 | 0.030 | 0.863 |
| IL5 |  | 0.084 | 0.772 | 0.244 | 0.622 | 1.105 | 0.293 | 2.084 | 0.149 | 0.983 | 0.321 | 0.000 | 0.986 | 0.075 | 0.785 |
| IL10 |  | 2.675 | 0.102 | 0.027 | 0.870 | 0.414 | 0.520 | 1.621 | 0.203 | 0.001 | 0.979 | 0.041 | 0.840 | 0.230 | 0.631 |
| IFNG |  | 0.319 | 0.572 | 0.000 | 0.991 | 4.515 | 0.034 | 6.303 | 0.012 | 0.115 | 0.735 | 0.925 | 0.336 | 2.020 | 0.155 |
| FOXP3 |  | 0.863 | 0.353 | 1.815 | 0.178 | 0.750 | 0.386 | 0.004 | 0.952 | 1.808 | 0.179 | 2.290 | 0.130 | 1.277 | 0.258 |

Table S11. The association between DNA methylation level of the *IL4*, *IL5*, *IL10*, *IFNG* and *FOXP3* loci and type of infant feeding. C – control group, A – allergic group, FA – group with food allergy, AD – group with atopic dermatitis, ADFA – group with atopic dermatitis and food allergy, H_K-W_ – Kruskal-Wallis ANOVA coefficient, level of significance p<0.05.
