# Supplementary material for: The methylation profile of IL4, IL5, IL10, IFNG and FOXP3 associated with environmental exposures differed between Polish infants with the food allergy and/or atopic dermatitis and without the disease
Source: Front Immunol. 2023 Jul 13;14:1209190. doi: 10.3389/fimmu.2023.1209190 (PMC10373304; doi:10.3389/fimmu.2023.1209190)
Supplement: Supplementary file 12 [file Table_12.docx]

| Locus | Variable | Control group | | Allergic group | | FA | | AD | | ADFA | | FA+ADFA | | AD+ADFA | |
| --- | --- | --- | --- | --- | --- | --- | --- | --- | --- | --- | --- | --- | --- | --- | --- |
|  |  | rho | p | rho | p | rho | p | rho | p | rho | p | rho | p | rho | p |
| IL4 | Mother’s weight | -0.155 | 0.157 | 0.082 | 0.362 | -0.054 | 0.766 | 0.279 | 0.355 | 0.015 | 0.891 | 0.030 | 0.748 | 0.076 | 0.469 |
| IL5 |  | -0.299 | 0.005 | 0.099 | 0.269 | 0.126 | 0.486 | 0.062 | 0.841 | 0.076 | 0.499 | 0.095 | 0.313 | 0.078 | 0.452 |
| IL10 |  | -0.062 | 0.571 | -0.019 | 0.830 | 0.111 | 0.540 | -0.072 | 0.814 | -0.085 | 0.453 | -0.008 | 0.935 | -0.090 | 0.390 |
| IFNG |  | -0.166 | 0.128 | -0.005 | 0.958 | -0.197 | 0.271 | 0.263 | 0.385 | 0.068 | 0.548 | -0.035 | 0.714 | 0.101 | 0.333 |
| FOXP3 |  | -0.030 | 0.782 | 0.021 | 0.818 | -0.135 | 0.452 | 0.291 | 0.336 | 0.022 | 0.847 | -0.023 | 0.808 | 0.077 | 0.461 |
| IL4 | Mother’s BMI | -0.174 | 0.110 | 0.048 | 0.594 | -0.083 | 0.646 | 0.061 | 0.842 | 0.022 | 0.849 | 0.030 | 0.750 | 0.031 | 0.765 |
| IL5 |  | -0.330 | 0.002 | 0.150 | 0.092 | 0.149 | 0.409 | -0.069 | 0.823 | 0.135 | 0.230 | 0.165 | 0.079 | 0.112 | 0.282 |
| IL10 |  | -0.095 | 0.385 | 0.038 | 0.671 | 0.163 | 0.365 | -0.135 | 0.660 | 0.002 | 0.988 | 0.057 | 0.548 | -0.018 | 0.866 |
| IFNG |  | -0.238 | 0.028 | 0.066 | 0.459 | -0.204 | 0.254 | 0.213 | 0.486 | 0.197 | 0.078 | 0.054 | 0.565 | 0.196 | 0.059 |
| FOXP3 |  | 0.010 | 0.925 | 0.012 | 0.894 | -0.063 | 0.726 | 0.275 | 0.363 | 0.024 | 0.832 | -0.012 | 0.902 | 0.050 | 0.632 |
| IL4 | Child’s birth weight | -0.080 | 0.453 | -0.012 | 0.886 | 0.141 | 0.397 | 0.015 | 0.959 | -0.037 | 0.734 | 0.004 | 0.961 | -0.048 | 0.637 |
| IL5 |  | -0.031 | 0.770 | -0.009 | 0.917 | -0.047 | 0.778 | -0.027 | 0.924 | 0.010 | 0.928 | -0.018 | 0.846 | 0.024 | 0.813 |
| IL10 |  | -0.128 | 0.233 | 0.203 | 0.017 | 0.232 | 0.161 | 0.146 | 0.603 | 0.176 | 0.107 | 0.205 | 0.023 | 0.182 | 0.070 |
| IFNG |  | -0.068 | 0.528 | 0.119 | 0.163 | 0.056 | 0.738 | 0.070 | 0.805 | 0.182 | 0.096 | 0.134 | 0.141 | 0.149 | 0.139 |
| FOXP3 |  | -0.070 | 0.512 | -0.145 | 0.090 | -0.046 | 0.784 | -0.297 | 0.283 | -0.194 | 0.075 | -0.135 | 0.135 | -0.197 | 0.050 |
| IL4 | Child’s current weight | -0.080 | 0.453 | -0.012 | 0.886 | 0.141 | 0.397 | 0.015 | 0.959 | -0.037 | 0.734 | -0.024 | 0.790 | -0.051 | 0.618 |
| IL5 |  | -0.031 | 0.770 | -0.009 | 0.917 | -0.047 | 0.778 | -0.027 | 0.924 | 0.010 | 0.928 | -0.045 | 0.621 | -0.071 | 0.490 |
| IL10 |  | 0.0128 | 0.528 | 0.203 | 0.017 | 0.232 | 0.161 | 0.146 | 0.603 | 0.176 | 0.107 | 0.109 | 0.233 | 0.155 | 0.128 |
| IFNG |  | -0.068 | 0.528 | 0.119 | 0.163 | 0.056 | 0.738 | 0.070 | 0.805 | 0.182 | 0.096 | 0.040 | 0.663 | 0.129 | 0.205 |
| FOXP3 |  | -0.070 | 0.512 | -0.145 | 0.090 | -0.046 | 0.784 | -0.297 | 0.283 | -0.194 | 0.075 | 0.022 | 0.810 | -0.056 | 0.587 |

Table S12. The association between DNA methylation level of the *IL4*, *IL5*, *IL10*, *IFNG* and *FOXP3* loci and BMI/weight of mother and weight of infant. C – control group, A – allergic group, FA – group with food allergy, AD – group with atopic dermatitis, ADFA – group with atopic dermatitis and food allergy, rho – Spearmans’ rho coefficient, level of significance p<0.05.
