# Supplementary material for: The methylation profile of IL4, IL5, IL10, IFNG and FOXP3 associated with environmental exposures differed between Polish infants with the food allergy and/or atopic dermatitis and without the disease
Source: Front Immunol. 2023 Jul 13;14:1209190. doi: 10.3389/fimmu.2023.1209190 (PMC10373304; doi:10.3389/fimmu.2023.1209190)
Supplement: Supplementary file 13 [file Table_13.docx]

| Locus | Variable | Control group | | Allergic group | | FA | | AD | | ADFA | | FA+ADFA | | AD+ADFA | |
| --- | --- | --- | --- | --- | --- | --- | --- | --- | --- | --- | --- | --- | --- | --- | --- |
|  |  | rho | p | rho | p | rho | p | rho | p | rho | p | rho | p | rho | p |
| IL4 | Number of family members with allergy | 0.226 | 0.033 | 0.163 | 0.057 | 0.101 | 0.547 | 0.585 | 0.022 | 0.096 | 0.380 | 0.080 | 0.379 | 0.202 | 0.044 |
| IL5 |  | -0.015 | 0.888 | -0.025 | 0.767 | -0.210 | 0.205 | 0.292 | 0.291 | 0.063 | 0.570 | -0.052 | 0.571 | 0.063 | 0.535 |
| IL10 |  | -0.002 | 0.986 | -0.140 | 0.102 | -0.233 | 0.159 | -0.220 | 0.432 | -0.081 | 0.460 | -0.123 | 0.176 | -0.113 | 0.261 |
| IFNG |  | 0.059 | 0.586 | 0.002 | 0.978 | -0.065 | 0.696 | 0.436 | 0.104 | -0.071 | 0.521 | -0.054 | 0.556 | 0.015 | 0.884 |
| FOXP3 |  | 0.126 | 0.238 | 0.014 | 0.872 | -0.101 | 0.548 | 0.414 | 0.125 | -0.058 | 0.600 | -0.049 | 0.590 | 0.042 | 0.679 |

Table S13. The association between DNA methylation level of the *IL4*, *IL5*, *IL10*, *IFNG* and *FOXP3* loci and number of family members with allergy. C – control group, A – allergic group, FA – group with food allergy, AD – group with atopic dermatitis, ADFA – group with atopic dermatitis and food allergy, rho – Spearmans’ rho coefficient, level of significance p<0.05.
